# Is a single model enough? The systematic comparison of computational approaches for detecting populist radical right content

**DOI:** 10.1007/s11135-024-02034-1

**Published:** 2025-01-29

**Authors:** Mykola Makhortykh, Ernesto de León, Clara Christner, Maryna Sydorova, Aleksandra Urman, Silke Adam, Michaela Maier, Teresa Gil-Lopez

**Affiliations:** 1 Institute of Communication and Media Studies, University of Bern, Fabrikstrasse 8, 3012 Bern, Switzerland; 2 Institute for Communication Psychology and Media Education, University of Kaiserslautern-Landau, Landau, Germany; 3 Social Computing Group, University of Zurich, Zurich, Switzerland; 4 Department of Communication, University Carlos III of Madrid, Getafe, Spain

**Keywords:** Automated content analysis, Populist radical right, Supervised machine learning, Neural networks, Dictionaries, Ensemble modeling

## Abstract

The rise of populist radical right (PRR) ideas stresses the importance of understanding how individuals engage with PRR content online. However, this task is complicated by the variety of channels through which such engagement can take place. In this article, we systematically compare computational approaches for detecting PRR content in textual data. Using 66 dictionary, classic supervised machine learning, and deep learning (DL) models, we compare how these distinct approaches perform on the PRR detection task for three Germanophone test datasets and how their performance is affected by different modes of text preprocessing. In addition to individual models, we examine the performance of 330 ensemble models combining the above-mentioned approaches for the dataset with a particularly high volume of noise. Our findings demonstrate that the DL models, in combination with more computationally intense forms of preprocessing, show the best performance among the individual models, but it remains suboptimal in the case of more noisy datasets. While the use of ensemble models shows some improvement for specific modes of preprocessing, overall, it mostly remains on par with individual DL models, thus stressing the challenging nature of computational detection of PRR content.

## Introduction

The growing support for populist radical right (PRR) ideas around the world and the threats they pose for liberal democracies (e.g., Bornschier 2018; Muis and Immerzeel 2017) stresses the importance of understanding how individuals are exposed to these ideas. Under the conditions of the high-choice media environment (van Aelst et al. 2017), such exposure can occur across a wide range of platforms, thus complicating the task of studying it. While new methods of data collection (e.g., web-tracking data; Christner et al. 2022) enable novel possibilities for research on exposure to PRR ideas, the volume and variety of content circulating PRR ideas prompt the need for developing computational approaches for such content's detection.

The ability to detect PRR content based on the complex PRR construct is important for several reasons. The ongoing rise of PRR parties, in particular across Europe, results in dramatic changes in the communication landscape by contributing to the increased focus on conflicts and scandals in the media sphere (Muis and Immerzeel 2017). While there are left-leaning populist movements, the thin ideology of populism is of particular concern when connected with the elements of right-wing ideologies (e.g., nativism and authoritarianism). Under these circumstances, being able to trace the engagement with content including features of populism, authoritarianism, and nativism in online environments is important for understanding how the rise of PRR ideas transforms the public sphere and how well-grounded the concerns regarding the negative consequences of such a transformation for the liberal democracies are.

In addition to studying exposure to PRR ideas, PRR content detection can advance research and facilitate policy-making in several other areas. Considering the frequent engagement of PRR actors with conspiratorial ideas and disinformation narratives (for instance, in the case of Russia’s war against Ukraine; Ivaldi & Zankina, 2023), the development of PRR content detection approaches can contribute to better understanding of the emergence and the spread of other forms of harmful content in online environments. Similarly, because PRR ideas often fuel different forms of hate speech, PRR content detection is important for studying the evolution of hate speech discourses and informing policy responses for their regulation. It can also contribute to assessing communicative practices emerging in different online environments and exploring the boundaries between civil and uncivil politics-related communication.

The ability to detect PRR content automatically on a large scale and across platforms is also important to be able to detect the messages of a broad range of populist actors (including potentially emerging figures) aiming to manipulate public opinion and mobilize their supporters; additionally, it enables new possibilities for cross-platform PRR campaign analysis. Finally, the computational detection of PRR content enables more opportunities for studying the effects of this content on individual behavior, in particular when coupled with novel approaches for systematic online behavior monitoring (for instance, web tracking; Christner et al. 2022). The latter approaches can also provide possibilities for achieving a better understanding of the role of algorithmic systems such as search engines and algorithmic news feeds in the spread and potential amplification of the visibility of PRR content: considering the often stochastic nature of these systems, the ability to reliably detect PRR content on a large scale is essential for systematic analysis of interactions between algorithms and PRR ideas.

Despite the growing interest in the computational detection of PRR content (e.g., Gründl 2020; Pauwels 2017; Ulinskaitė and Pukelis 2021), there are several reasons why it is hard to realize empirically. The first of these reasons is the complex nature of PRR ideas which are a construct made of multiple components (i.e., populism, nativism, and authoritarianism; Mudde 2007). Under these circumstances, scholars either have to use separate detection models for individual PRR components (e.g., populism) or aim to detect PRR content as a complex construct. Currently, most existing research focuses on the detection of a single PRR component (i.e., populism; Gründl 2020; Pauwels 2017), whereas the use of computational approaches for detecting other components as well as PRR content as a construct remains under-studied.

Another reason is related to the above-mentioned variety of content which can be used for circulating PRR ideas. This variety results in a large volume of noise (Agarwal et al. 2007), which can decrease the performance of detection models trained on a specific type of content (e.g., party documents; Ulinskaitė and Pukelis 2021) if these models are applied to other types of content (e.g., social media posts). The noise problem is amplified by the growing availability of large volumes of multi-platform textual data, which require extensive preprocessing to reduce the amount of noise (e.g., in the form of HTML artifacts left after parsing). However, the issue of noise and the ways to deal with it currently remain under-investigated, with existing studies usually focusing on PRR content detection in a single type of documents with low amounts of noise, such as political manifestos (Pauwels 2011; Ulinskaitė and Pukelis 2021).

In our study, we aim to address some of the above-mentioned challenges by conducting a methodological inquiry into how different types of automated approaches perform regarding PRR content detection. While doing so, we follow Blassnig et al. (2019) in treating populism—and PRR ideas more broadly—as a fragmented concept. According to Mudde (2007), PRR ideas are constituted by the combination of authoritarianism, nativism, and populism, albeit based on existing empirical research, textual material appearing online is likely to present not it as “as a thoroughly elaborated ideology, but as a collection of fragments” (Blassnig et al. 2019: 634). While initially, we aimed to operationalise the detection of PRR fully following the definition of Mudde—i.e., as content containing simultaneously elements of authoritarianism, nativism, and populism—we encountered substantive difficulties in finding such occurrences in the smaller units of texts coming from online platforms which we used as training data for our study. Under these circumstances, we opted for a more limited treatment of PRR ideas as a fragmented concept, assuming that appearance of individual PRR elements can be indicative of the presence of the PRR concept.

Such a treatment shall be taken into consideration when evaluating the contribution of the current study, in particular in the light of the ongoing discussions of challenges associated with the proper operationalization of complex political constructs (e.g., populism; Jankowski & Huber 2023) for automated content analysis and the validation of such analytical approaches. Due to limitations in training data, our study can not be treated as a study detecting PRR content as an ideal type; instead, we rely on the training dataset made of sentences associated with individual PRR components and use the predictions enabled by it as a proxy for detecting the presence of PRR ideas. Under these conditions, we see our main contribution as advancing research on PRR content detection beyond attempts to automatically detect populism by considering possibilities to detect other components of PRR (e.g., authoritarianism and nativism) and demonstrating the importance of diversifying the process of validating PRR-related automated content detection approaches through the integration of different datasets (thus, aligning with the important points made by Jankowski and Huber 2023).

For this aim, in this methodological paper, we systematically compare the performance of computational approaches for PRR content detection in Germanophone textual data, which rely on the operationalization of PRR ideas as a fragmented multi-component construct. Such comparison is important due to the rapid expansion of the computational approaches that are used for the automated detection of different forms of politics-related content. Using a large number of individual (n = 66) and ensemble (n = 330) models, we compare the performance of three groups of detection approaches—dictionaries, classic supervised machine learning (SML), and deep learning (DL)—across three test datasets with varying degrees of noise. By doing so, we also discuss how the performance of the low-cost implementations of these techniques is affected by different modes of text preprocessing.

This paper is organized as follows. First, we provide an overview of existing research on the computational approaches to PRR content detection and the possible impact of text preprocessing on these approaches. Then, we introduce the methodology used to develop individual and ensemble models for PRR content detection and compare their performance. It is followed by our findings on the performance of detection models across three test datasets and how it is affected by different modes of preprocessing. Finally, we discuss the implications of our findings for the use of computational approaches for PRR content detection, together with the limitations of the study and directions for future research.

## Related work

### Automated detection of PRR content

A growing body of scholarly research has looked at the detection of ideological leaning in textual data. A number of studies (e.g., Engesser et al. 2017; Ernst et al. 2017; Mazzoleni and Bracciale 2018, Fernández-García and Salgado, 2020) applied qualitative or mixed-method approaches to study different forms of populist content in online environments, ranging from politicians’ statements to social media users’ comments. The main advantage of these approaches relates to their ability to capture nuanced features of PRR content which can not necessarily be identified through the automated means, in particular considering the dependency of computational approaches on the data used to develop them (e.g., training datasets for classic SML and DL models). Considering that it is close to impossible to establish a training dataset capturing the whole breadth of PRR content and accounting for the evolution of such content over time, the validity of properly implemented qualitative approaches for PRR content detection is inevitably higher than that of computational ones. However, the major limitation of qualitative approaches regards their limited scalability—i.e., it is difficult to qualitatively detect PRR content for datasets exceeding several thousands of web pages, and many datasets (e.g., the ones established with the help of data donations or web tracking) can consist of millions of URLs and the corresponding web pages.

To address the limited scalability of the qualitative content analysis approaches in the context of studying populist communication, many recent studies have applied advances in the fields of SML and DL. These studies looked at tasks ranging from the detection of biased coverage in the news (Budak et al. 2016; Gentzkow and Shapiro 2010; Kulkarni et al. 2018) to the analysis of linguistic differences in politicians’ speeches (Diermeier et al. 2012; Iyyer et al. 2014). However, the approaches for detecting more specific forms of ideological content, particularly those dealing with PRR elements, remains less studied.

One exception in this context is the detection of populism which can be viewed not only as an ideology but also as a communicative style (Gründl 2020). For a long time, research on the detection of populism in textual data relied on manual content analysis (Ernst et al. 2017, 2019; Jagers and Walgrave 2007; Hawkins 2009) and tended to focus on political texts disseminated via analog means (e.g., political manifestos; Jagers and Walgrave 2007; Hawkins 2009). However, in recent years, a growing number of studies have looked at how populist content is propagated in digital media (e.g., social media posts; Ernst et al. 2017, 2019).

Studies relying on automated approaches for the detection of populist content primarily utilize dictionary-based approaches that use self-constructed dictionaries and apply them to political party documents (e.g., manifestos). Pauwels (2011), for instance, analyzed internal magazines of Belgian political parties and their manifestos using a self-constructed dictionary of terms related to central populist themes. A similar approach, but with a dictionary focused on anti-elitism vocabulary (established according to theoretical assumptions and empirical examination of European populist parties’ manifestos), was used by Rooduijn and Pauwels (2011) to conduct a comparative detection of populism in parties’ documents. The comparison of the dictionary-based approach and manual content analysis in the latter study showed that the automated approach performed worse but required substantially less resources and time to be implemented.

The application of dictionary-based approaches for populism detection outside political parties’ documents currently remains limited. Gründl (2020) built on existing dictionaries (i.e., from Rooduijn and Pauwels (2011) and Pauwels (2017)) and expanded them using theoretical considerations and synonyms to detect populism in Facebook and Twitter content published by Swiss, Austrian, and German political parties. While the resulting approach was not evaluated on a hand-coded dataset of texts, its performance was assessed conceptually by correlating its assessment of populist parties’ communication with populist scores assigned in expert surveys, resulting in a correlation of 0.83 (compared to 0.74 for the dictionary from Rooduijn and Pauwels (2011) and 0.63 for the dictionary from Pauwels (2017)). Another example is the study by Hameleers and Vliegenthart (2020) on the detection of populism in journalistic content, in which they developed a populism dictionary based on people centrism, anti-elitism, and right- and left-wing exclusionism. Here, a random sample of 100 documents coded as populist revealed relevance scores of above 90% for each component.

While dictionary-based approaches are prevalent, there are also several studies utilizing SML and DL approaches to detect populism. For instance, Ulinskaitė and Pukelis (2021) used a pre-trained Bidirectional Encoder Representations from Transformers (BERT) model to detect populism in the manifestos of two Lithuanian parties from 2016 to 2020. They used a database of hand-coded manifestos to provide training material for the models. While manifestos were originally written in Lithuanian, for the purposes of the task, they were automatically translated into English and split into paragraphs. Their results revealed a performance of F1 scores between 0.2 (a k-nearest neighbors approach) and 0.79 (an ensemble approach), with the highest score being produced by an ensemble of two models. Hawkins and Silva (2018) used the penalized least squares method with an elastic net penalty to detect populism in manifestos of political parties from 27 countries in Western Europe and the Americas, using hand-coded manifestos as the source of training; similar to Ulinskaitė and Pukelis (2021), the manifestos were also translated to English automatically. The penalized least squares method allows optimizing models to provide the best fit to the data without allowing models to become too complex.

The automated detection of the other two elements of PRR—nativism and authoritarianism—has received far less attention. Work on detection of migration-related content indirectly informs the identification of nativist content. Studies have qualitatively coded anti-immigration frames in tweets (Walsh 2021), as well as used automated approaches such as principal component analysis (Blinder and Allen 2016; Greussing and Boomgaarden 2017) and dictionaries (Woods and Arthur 2014) to automatically identify media frames regarding the topic of migration, including prominent “security threat” frames (for a review, see Eberl et al. 2018). While not explicitly looking at nativist content, these studies capture the anti-immigration rhetoric that is central to nativism using both qualitative content labeling and computational text analysis.

Similarly, research on authoritarianism has been limited to date. As a notable exception, Perrin (2005) manually coded the presence of authoritarianism in letters to the editors following the September 11 attacks in the United States. The study made use of a coding scheme that distinguished between various elements of authoritarian expression in the text, including the expression of power and toughness, authoritarian aggression, and authoritarian submission, as well as several anti-authoritarian codes. Collapsing these into broader expressions of pro- and anti-authoritarianism, Perrin argued that the shifting political climate could be gauged in these texts, with increased support for authoritarianism.

This study is an exception to most studies on authoritarianism that treat the concept as an attitude or a personality trait (Adorno et al. 1950), while Perrin argues that authoritarianism “can be identified by the mode of argument and the tendency to repress, censor, or punish others” (Perrin 2005: 168), allowing for its identification in texts. Other terrorism-related content analyses—where support for the securitarian state and the sacrifice of civil rights are detected—discuss their findings in relation to authoritarianism (e.g., Bogain 2020), but rarely focus on detecting the authoritarianism itself in texts. Research on social movements also provides some efforts to tackle authoritarianism-related content detection; these studies, however, tend to focus on anti*-*authoritarianism content in the media and social media (e.g., Yildiz and Smets 2019), usually through manual labeling.

### Preprocessing and automated content analysis

Preprocessing decreases the amount of noise in textual data by reducing the complexity of textual features (Grimmer and Stewart 2013), which is particularly important for dealing with multi-platform data, where the amount of noise is higher compared with monoplatform data. Some basic modes of preprocessing include letter lowercasing, punctuation removal, and the exclusion of repeating characters (HaCohen-Kerner et al. 2020). More complex modes deal with stopword removal (i.e., the removal of very common words or those with little meaning such as “the”); stemming, where words are stripped to their base by removing verb and adverb suffixes (e.g., “ed” and “ly”); and lemmatization, where inflicted versions of words are converted to their neutral lemma (e.g., “am” and “is” become “be”).

Despite the increasing number of studies conducting systematic analyses of the effects of preprocessing on the performance of automated content analysis approaches (e.g., Denny and Spirling 2018; HaCohen-Kerner et al. 2020), the choice of an optimal mode of preprocessing remains a challenging task. Its complexity can be attributed to several factors. First, the majority of studies compare the impact of preprocessing within a specific group of content detection approaches (e.g., SML; HaCohen-Kerner et al. 2020) and rarely examine the variation between different groups of techniques (e.g., classic SML and DL). Second, while the effects of preprocessing are influenced by the contextual factors associated with the task (e.g., the language of the dataset), most research focuses on Anglophone textual data, thus limiting the possibilities for investigating the impact of these factors. Third, there can be multiple implementations of the same preprocessing mode; while the impact of such differences can be marginal (e.g., different stemmers resulting in less than a 0.01 difference in accuracy scores; Bounabi et al. 2019), it nevertheless causes variation in the approach’s performance.

Under these circumstances, many studies argue for the use of less complex modes of preprocessing that require fewer computational resources but nevertheless improve performance. For instance, HaCohen-Kerner et al. (2020) compared the impact of simpler forms of preprocessing on the performance of three SML models for Anglophone data and found that stopword removal resulted in the most consistent performance improvement for two out of three of the models. Similarly, the beneficial effect of stopword removal was observed in a study examining the effects of preprocessing on SML-based approaches for Czech data (Toman et al. 2006). However, in some cases, such as when dealing with corpora with few stopwords (e.g., spam mails; Méndez et al. 2005), the removal of stopwords actually worsened the model performance.

Compared with simple modes of preprocessing, such as stopword removal or the reduction of repeated characters, more complex modes (e.g., lemmatization) enable more feature reduction and thus can provide larger performance increases for approaches affected by data noise (e.g., SML-based ones; Denny and Spirling 2018). In practice, however, the effect of complex modes of preprocessing turns to be rather ambiguous: in some cases, particularly those dealing with SML, stemming (Gonçalves et al. 2010) or lemmatization with stopword removal (El Kah and Zeroual 2021) resulted in performance improvements. In other cases, these modes of preprocessing resulted in marginal improvements (Song et al. 2005) or an actual drop in performance (Toman et al. 2006).

This ambiguous effect of more complex forms of preprocessing is particularly pronounced in the case of DL-based approaches, which sometimes benefit from higher noise (e.g., in the form of stopwords) that enables more possibilities to understand contextual relationships within the corpus. Maulana and Maharani (2021) showed that in the case of disaster-related Twitter content in English, stemming and stopword removal led to a decrease in the performance of the BERT model because of the elimination of text features. At the same time, Konstantinov et al. (2020) found that for Russian language computational tasks using BERT, lemmatization enabled performance improvement, whereas stemming did not.

## Methodology

### Operationalizing PRR for its computational detection

Following Mudde (2007), we initially aimed to treat PRR as a complex construct made of three components: authoritarianism, nativism, and populism (for more information on our definition of individual components and their operationalization for the preparation of the training and test datasets, see Appendix A1). Based on this treatment, the content item shall include features related to these three components in order to be viewed as PRR. To evaluate whether this is the case, we would need either to detect each component separately (and then assign a PRR label if all three models—i.e., one per individual PRR component—agree that their respective component is present) or detect PRR on the aggregate level using training data with features of all three components (and then assign a PRR label if the aggregate-level model detects presence of PRR).

For this paper, we opted for the second option with substantive limitations attributed to how PRR content appears in online environments and, consequently, in online environment-based training data, which we outline in more detail below and in the discussion section. We decided to do so for a number of reasons. First, this option is less resource-consuming, in particular when working with large volumes of textual data with a high volume of noise (for more information on our use case, see Appendix A2). Second, content items associated with individual PRR components were unequally distributed in our training dataset (see below) and it limited our possibilities for developing component-specific detection models. This unequal distribution can be attributed to the various presence of individual PRR components within PRR content. Under these conditions, we opted for proceeding with the detection of PRR based on the assumption that it will appear as a “fragmented” (Blassnig et al. 2019: 635) concept. While such an operationalization does not match the ideal type operationalization of PRR as a multi-dimensional construct simultaneously combining features of populism, authoritarianism, and nativism, we still believe that it can serve as a proxy for capturing PRR content (albeit the generalizability of such approach can be questioned). Specifically, we tested this approach by re-arranging our training datasets to construct larger chunks of text (e.g., made of eight sentences) to assign the PRR label only under the condition where all three PRR components appear together in the same chunk of texts, and then applying some of trained model to evaluate their performance on these new datasets which were closer to capturing PRR as a multi-dimensional construct. Our findings showed drops in performance for the updated train-test split test dataset, whereas performance for the updated sentence-based test dataset slightly improved; these observations highlight that our approach can also to a certain degree capture PRR based on its ideal type operationalization.

Similar to other studies dealing with computational approaches for content detection (e.g., Barberá et al. 2021), we also had to decide whether to detect PRR content on the document or the sentence level. Initially, we aimed at the document level following earlier research on politics-related content detection (Makhortykh et al. 2022). However, the manual labeling of training data demonstrated that it is difficult for the coders to detect PRR content on the document level. Even content items coming from right-wing hyperpartisan outlets (e.g., Journalistenwatch) included relatively few statements explicitly referring to PRR ideas. Under these circumstances, we presumed that focusing on the sentence-level detection would facilitate the identification of relevant features by the PRR content detection models.

### Training dataset

To produce the training dataset, we used a structured random sample of web pages captured in the course of the project focused on studying the exposure of German and Swiss citizens to PRR content. We sampled pages coming from right-wing hyperpartisan outlets, which we assumed to be likely to include PRR content together with pages from non-hyperpartisan outlets and containing politics-related information^1^ (see Appendix A1 for more information). We used the selectolax Python library (Golubin 2022) to extract text from pages’ HTML and the NLTK library (Bird et al. 2009) to split it into sentences.

Altogether, we extracted 27,430 unique sentences from 757 web pages. Then, a team of five coders was trained to detect the presence of PRR content in the sentences. We conducted six rounds of training until satisfactory reliability values were achieved (Table 1). We used Krippendorff’s Alpha as a measure of intercoder reliability and the Holsti coefficient as a measure of intercoder agreement. 2689 sentences were labeled as non-codable (e.g., because of being too short or malformed as a result of text-from-HTML extraction), and 2,140 sentences were labeled as the ones containing PRR content. The remaining 22,601 sentences did not contain PRR content.

**Table 1:** Intercoder reliability for preparing the training dataset based on the subset (N = 20) of sentences

PRR component	Krippendroff’s alpha	Holsti coefficient
Authoritarianism	0.67	0.88
Nativism	0.63	0.90
Populism	0.78	0.91

Within 2,140 sentences containing PRR content, the distribution of individual PRR components was unequal. 781 sentences contained content related to nativism, 944—to authoritarianism, and 415—to populism.

### Individual PRR content detection models

The training dataset was used to develop 11 PRR content detection models using dictionary (three models), SML (five models), and DL (three models) approaches. These models were combined with six different modes of preprocessing (see below), resulting in 66 models. All SML models and dictionaries are available via the OSF repository (https://osf.io/asuwg/; folder "Models for PRR content detection" and subfolders "CSML" and "Dictionaries"). Due to their size, DL models are available via the Harvard Dataverse (10.7910/DVN/SPL509).

The dictionary approach involved three models, which we labeled as Di-Gr, Di-LL, and Di-Gr-LL. The Di-Gr model was based on the adapted version of the dictionary developed for the analysis of populist content in German social media by Gründl (2020). The Di-LL model used a dictionary constructed from the training dataset using the log-likelihood keyword analysis (Pojanapunya and Todd 2018) to identify terms that were overrepresented in the sentences containing PRR content. Following existing studies (e.g., de Schryver 2012), we created two sub-dictionaries consisting of the top 100 and 1,000 terms (according to log-likelihood scores) and then compared their performance across three test datasets (using no preprocessing mode; see below). Based on the average F1 scores across the test datasets, the 100-term option demonstrated better performance and was thus used in the study. Finally, the Di-Gr-LL model combined the dictionary developed by Gründl with the log-likelihood-based dictionary. The assumption here is that the combined dictionary might outperform its components by bringing together the theoretically informed set of terms (Di-Gr) and the empirically driven set of terms that were most common in PRR-related sentences (Di-LL).

Unlike SML and DL approaches, dictionaries do not provide a binary label (i.e., whether a sentence contains PRR content or not). Instead, dictionaries detect the number of terms within a sentence related to a specific construct such as PRR and this number then has to be translated into a binary label. The translation can be based either on the absolute number of the PRR-related words or the ratio between the number of PRR-related words to the overall number of terms per sentence. Because of our focus on sentence-level detection, we decided to opt for the absolute number of words which we expected to be a more robust measure due to the artificial inflation of ratios for very short sentences.

To decide on the optimal threshold of the number of unique PRR-related words per sentence, we calculated their occurrences per each sentence in the two test sets (i.e., the train-test split test dataset and sentence-based test dataset; see below for more information). Then, we used each unique number of occurrences (e.g., one word per sentence or five words per sentence) as a possible threshold for assigning a PRR label to all the sentences in the respective test dataset and calculated the resulting average F1 scores. Then, we chose the thresholds that achieved the maximum F1 score for the respective test datasets (i.e., one threshold per dataset) and applied them to another dataset to identify a single threshold with the most consistent performance. This procedure was repeated for each dictionary-based technique and for each of the six modes of preprocessing. The complete list of optimal thresholds is provided in Appendix A5, but in most cases, the best performance was achieved with the threshold of at least two PRR-related words per sentence.

For SML, we trained five models: Bernoulli naive Bayes (BNB), multinomial naive Bayes (MNB), logistic regression (LR), passive aggressive (PA), and stochastic gradient descent (SGD). These models differ in complexity, with some being based on simple Bayesian probabilistic modeling (e.g., BNB) and others relying on more advanced incremental learning principles (e.g., PA). All SML models were trained using the Scikit-Learn package for Python (Pedregosa et al. 2011).

For DLs, we used three models: a convolutional neural network (CNN), a long short-term memory (LSTM) network, and BERT. CNNs have low computational costs compared with other types of neural networks and focus on high-level features, while LSTM networks place major emphasis on term sequences. Finally, BERT (Devlin et al. 2019) is a transformer model characterized by high computational costs but also more advanced capabilities for processing sequential data, along with an extensive awareness of contextual relationships between words attributed to it being pre-trained on a large text corpus.

To train the CNN and LSTM models, we used the Python Tensorflow library (Abadi et al. 2016), whereas, for BERT, we relied on a pre-trained model for German from HuggingFace (2020). Because of our interest in low-cost detection approaches, we used simple CNN and LSTM architectures (see Appendix A3) with five learning epochs and 256 embedding dimensions. For BERT, we used three epochs because of the higher computational costs of the model’s training and tested a series of probabilities for the “PRR” label to be assigned. Based on the F1 scores achieved per probability, we opted for 0.15 probability, which resulted in the highest F1 score.

### Ensemble PRR content detection models

Based on 66 individual models, we developed 330 ensemble models (i.e., 11 × (11–1)/2 models × 6 modes of preprocessing). The concept of the ensemble model is based on the assumption that individual computational models use different principles to generate output (e.g., a prediction of whether a content item is related to PRR ideas). Instead of relying on a single principle, ensemble models combine the strong sides of individual models by training several of them for the same task and under similar conditions (Ardabili and Várkonyi-Kóczy 2019). Then, the scores produced by each individual model serve as inputs for making a final prediction. The potential of this approach for improving the performance of complex natural language processing tasks is demonstrated by earlier studies (e.g., Ulinskaitė and Pukelis 2021) applying ensemble models for political communication research.

For our implementation of ensemble models, we combined predictions made by 66 individual models pairwise to evaluate the combined performance of each possible combination. While doing so, we relied on the inner join principle, where only those content items for which both models agreed were treated as the ones containing PRR content. While it would also be possible to use the outer join principle (i.e., where it is enough for at least one model to assign the PRR label to the item in order for it to be treated as the one containing PRR content), the results of our short non-systematic examination demonstrated that it worsens the performance, as most models suffered primarily from low precision.

Because the performance of individual models was particularly poor on the document-based test dataset and due to our use case (see Appendix A2) being focused on the detection of PRR content within documents, we decided to test the performance of ensemble models on the document-based test dataset as well as to examine how different modes of preprocessing influence it. In the Results section, we present the F1 scores for only the PRR class because it is where individual models showed particularly low performance. In the following figures, these combinations are visualized, including each model’s individual score (e.g., the BERT & BERT combination) for easy comparison. In Appendix A7, the scores are provided for all three classes (i.e., PRR, non-PRR, and the macro average score).

### Data preprocessing

As a starting point, we lowercased all words in the training and test datasets to avoid potential inconsistencies and removed punctuation marks using the Python String package (Python 2022). Then, we systematically compared models’ performances across six preprocessing modes: (1) no additional preprocessing; (2) stopword removal (using the list of stopwords for German from the Python NLTK library; Bird et al. 2009); (3) stemming (using the Cistem stemmer from the NLTK library; Bird et al. 2009); (4) lemmatization (using the lemmatizer for German from the SpaCy library; Honnibal et al. 2020); (5) stopword removal + stemming; and (6) stopword removal + lemmatization.

### Datasets for evaluating models’ performance

To evaluate the models’ performance, we used three test datasets. Data for all three datasets came from the collection of web pages visited by the German and Swiss users (see Appendix A2) and included: (1) a *train-test split test dataset* made up of a subsample of training data (4,782 sentences; 20% of the training dataset) and constructed in a proportional manner to maintain the ratio between the PRR and non-PRR sentences; (2) *sentence-based test dataset* composed of 300 sentences selected using structured random sampling (i.e., 150 sentences from the pages coming from right-wing hyperpartisan outlets and 150 sentences from the pages coming from the broad range of platforms and containing politics-related information; (3) *document-based test dataset* made of 192 web pages randomly sampled from the subset of tracked pages containing politics-related information.

The manual labeling of the presence of PRR content for the train-test split test dataset followed the same principles as the preparation of the training dataset discussed above. The proportional sampling of PRR and non-PRR content from the training dataset resulted in the train-test split test dataset consisting of 4,394 non-PRR sentences and 388 PRR sentences. In the case of the sentence-based test dataset and the document-based test dataset, manual content labeling was done by the two coders and double-checked by the two experts working with PRR content in Germany and Switzerland. The process of labeling resulted in the sentence-based test dataset containing 134 PRR and 166 non-PRR sentences and the document-based test dataset containing 176 non-PRR documents and 16 PRR documents.

It is important to note a difference between the train-test split test dataset/document-based test dataset and the sentence-based test dataset in terms of the distribution of PRR and non-PRR content items. Unlike the sentence-based test dataset, where the ratio of PRR to non-PRR items is relatively close (i.e., 44–56%), the train-test split test dataset and the document-based test dataset have a strong prevalence of non-PRR items.

There are two other differences between the three test datasets. The first of them regards the unit of the analysis for automated PRR content detection: i.e., the sentence or the document. Our decision to include both sentence (train-test split test dataset and sentence-based test dataset) and document level (document-based test dataset) test datasets is attributed to two reasons. First, despite our decision to focus on the sentence-level detection of PRR content, we expected that there would be a need to translate the approach to the document level. This expectation is attributed to the document being a common unit of analysis for many detection tasks, including the ones dealing with PRR. Hence, for the document-based test dataset, we implemented the procedure for transferring the sentence-level detection to the document level, which we describe in more detail in the next section.

Second, we were interested in the models’ performance under the condition of the different degrees of noise. Understood as “difference in the surface form of an electronic text from the intended, correct or original text” (Agarwal et al. 2007: 5), noise is an important factor for content detection tasks, in particular with dealing with textual data coming from different platforms and in different formats. Considering the multi-platform nature of novel forms of exposure-related data (e.g., the ones provided via web-tracking; Christner et al. 2022), we find it important to account not only for scenarios when data have little noise but also the cases when the amount of noise can be high.

Among the three test datasets, we expect the amount of noise to be the lowest for the train-test split test dataset, which is made of the same content that is used to train the models. A low amount of noise characterizes the sentence-based test dataset because it is made of sentences (i.e., the same unit of analysis for which the models were trained) and is thoroughly preprocessed. Finally, the document-based test dataset has a high amount of noise being made of a diverse set of documents (i.e., a different unit of analysis) from which we extracted text using a universal HTML parser^2^ (based on the Selectolax Python library; Golubin 2022).

Based on our earlier research on the automated detection of politics-related content (Makhortykh et al. 2022), we expected that more complex SML and DL models would perform better on the test datasets containing a lesser amount of noise: i.e., train-test split and sentence-based test datasets. We also expected better performance from these models on the train-test split dataset, which is the closest to the data on which these models are trained, and it shall be beneficial considering the advanced capacities of SML and DL models to recognize patterns in the training data. By contrast, for more noisy data coming from the document-based test dataset, we would expect dictionaries to perform potentially better. Finally, we expect more similar performance of the models on the datasets with the same unit of analysis (train-test split and sentence-based test datasets) compared with the use of these models for document-level detection.

### Procedure for evaluating models’ performance

To compare the performance of models on the test datasets, we used three common evaluation metrics: precision, recall, and F1 score (Boumans and Trilling 2016). Precision is the ratio of true positive cases to the sum of true positives and false negatives, recall is the ratio of true positives to the sum of true positives and false positives, and the F1 score is the harmonic mean of precision and recall.

Because the F1 score combines two other metrics, we prioritized its reporting throughout the findings to demonstrate how models perform in terms of detecting the positive class (PRR content), the negative class (non-PRR content), and on average (i.e., between the two classes). Precision and recall for the individual models are reported in Appendix A6, whereas the metrics for the ensemble models are supplied as a separate dataset in the supplementary material due to the large number of values (i.e., over 600 rows).

It is important to note that no cross-validation was used when measuring the performance of the models, which made our observations about their performance less robust. This is a major limitation of the study that is attributed to the large number of models compared and the limited computational resources available, with the latter factor being particularly relevant for more computationally demanding approaches (e.g., the ones using BERT). Instead, we opted for a fixed train–test split to make the comparison between the models more consistent by ensuring that all models used the same data for training.

Finally, it is important to note that for the document-based test dataset we had to implement the transition between sentence-level and document-level  detection. For this aim, we calculated the ratio of sentences labeled as PRR to all sentences in each document within the document-based test dataset. Then, we implemented an iterative pipeline in which we calculated the F1 score for the whole document-based test dataset based on each of the 192 generated ratios and selected the one that showed the best performance (see Appendix A5). While doing so, we optimized for the average F1 score between the PRR and non-PRR classes.

## Results

### Individual PRR content detection models

Table 2 demonstrates the suboptimal performance of individual PRR content detection models on the train-test split test dataset. While the average F1 scores reached 0.73 (BERT), the F1 scores for the PRR class turned out substantially lower, with the highest score of 0.51 (BERT). Similar but slightly lesser scores were achieved by other DL (LSTM; F1 score of 0.42 for the PRR class) and SML models (LR; F1 score of 0.45 for the PRR class), whereas dictionary-based models performed quite poorly (maximum F1 score of 0.23 for the PRR class for Di-LL).

**Table 2:** Models’ performance on the train-test split test dataset

	No preprocessing	Stopwords	Stemming	Stemming + stopword removal	Lemmatizati on	Lemmatization + stopword removal
Di-Gr	0.17 [0.49]	0.17 [0.49]	0.14 [0.53]	0.16 [0.41]	0.16 [0.52]	0.15 [0.53]
Di-LL	0.23 [0.55]	0.20 [0.43]	0.20 [0.47]	0.20 [0.40]	0.22 [0.51]	0.20 [0.41]
Di-Gr-LL	0.22 [0.51]	0.19 [0.37]	0.20 [0.49]	0.21 [0.46]	0.20 [0.45]	0.22 [0.48]
SML [PA]	0.41 [0.68]	0.40 [0.68]	0.41 [0.68]	0.42 [0.69]	0.42 [0.69]	0.42 [0.69]
SML [BNB]	0.37 [0.66]	0.39 [0.67]	0.37 [0.66]	0.39 [0.67]	0.38 [0.67]	0.40 [0.68]
SML [MNB]	0.34 [0.65]	0.35 [0.65]	0.32 [0.64]	0.33 [0.64]	0.34 [0.65]	0.34 [0.65]
SML [LR]	0.40 [0.67]	0.45 [0.70]	0.41 [0.67]	0.42 [0.68]	0.40 [0.67]	0.43 [0.69]
SML [SGD]	0.38 [0.65]	0.2 [0.39]	0.37 [0.64]	0.36 [0.65]	0.39 [0.66]	0.37 [0.66]
DL [CNN]	0.40 [0.68]	0.28 [0.63]	0.40 [0.68]	0.38 [0.67]	0.38 [0.67]	0.37 [0.67]
DL [LSTM]	0.42 [0.69]	0.34 [0.66]	**0.42 [0.69]**	0.42 [0.69]	0.41 [0.68]	0.42 [0.69]
DL [BERT]	**0.48 [0.72]**	**0.49 [0.72]**	0.37 [0.62]	**0.45 [0.69]**	**0.51 [0.73]**	**0.45 [0.69]**

In this and the following tables, the highest performance values per preprocessing mode are bolded

The effects of preprocessing in the case of the train-test split test dataset had a limited impact on the models’ performance. While we observed differences between the performance of the models using no preprocessing and the ones using more computationally intensive modes (e.g., stemming or lemmatization), these differences usually were in the range of 0.01–0.06 for the F1 scores for the PRR class for the best performing models (e.g., BERT and LR). There were some exceptions related to the larger drop in performance of specific models associated with a specific mode of preprocessing (e.g., of 0.14 for stemming for BERT or 0.12 for stopword removal for CNN), but overall the impact of preprocessing was not large.

Compared with the train-test split test dataset, models' performance on the sentence-based test dataset (Table 3) improved substantially. Similar to the train-test split test dataset, BERT showed the best performance overall with the F1 score of 0.85 both on the average and for the PRR class, whereas other DL models (i.e., LSTM and CNN) experienced a drop in performance. SML models, in particular LR and SGD, again showed acceptable performance, with F1 scores for the PRR class reaching 0.72 and 0.71. Dictionaries, in particular Di-Gr-LL, demonstrated the second-best performance, with the F1 scores for the average and PRR class being in the range of 0.74–0.76.

**Table 3:** Models’ performance on the sentence-based test dataset

	No preprocessing	Stopwords	Stemming	Stemming + stopword removal	Lemmatization	Lemmatization + stopword removal
Di-Gr	0.58 [0.65]	0.48 [0.62]	0.33 [0.54]	0.62 [0.63]	0.39 [0.55]	0.39 [0.55]
Di-LL	0.62 [0.74]	0.39 [0.55]	0.68 [0.70]	0.73 [0.72]	0.67 [0.72]	0.73 [0.72]
Di-Gr-LL	0.64 [0.70]	0.48 [0.62]	0.67 [0.71]	0.70 [0.72]	**0.74 [0.75]**	**0.75 [0.76]**
SML [PA]	0.54 [0.66]	0.43 [0.6]	0.49 [0.64]	0.43 [0.6]	0.47 [0.62]	0.43 [0.6]
SML [BNB]	0.36 [0.55]	0.33 [0.54]	0.39 [0.57]	0.38 [0.56]	0.38 [0.57]	0.38 [0.56]
SML [MNB]	0.25 [0.5]	0.27 [0.51]	0.18 [0.45]	0.21 [0.48]	0.22 [0.48]	0.22 [0.48]
SML [LR]	**0.71 [0.77]**	0.65 [0.73]	0.68 [0.74]	0.67 [0.74]	0.72 [0.78]	0.70 [0.76]
SML [SGD]	0.68 [0.75]	**0.71 [0.65]**	0.68 [0.75]	0.52 [0.65]	0.64 [0.73]	0.59 [0.7]
DL [CNN]	0.35 [0.55]	0.35 [0.56]	0.35 [0.55]	0.35 [0.56]	0.33 [0.54]	0.33 [0.54]
DL [LSTM]	0.48 [0.62]	0.46 [0.61]	0.48 [0.62]	0.46 [0.61]	0.45 [0.61]	0.40 [0.58]
DL [BERT]	0.53 [0.66]	0.64 [0.71]	**0.85 [0.85]**	**0.75 [0.77]**	0.66 [0.73]	0.71 [0.75]

In terms of the impact of preprocessing, the sentence-based test dataset showed a larger variation between specific preprocessing modes compared with the train-test split test dataset. In the case of the sentence-based test dataset, preprocessing resulted in major differences in performance for the best-performing models. In the case of BERT, the difference between the F1 scores for the best-performing option (i.e., the one with stemming) and the least-performing one (i.e., the one without preprocessing) was 0.32. Similarly large was the difference for the second-performing option—i.e., Di-Gr-LL—where the difference in F1 score between the model using lemmatization and the model using stopword removal was 0.27.

Table 4 shows that the performance of models for the document-based test dataset was similar to the train-test split test dataset. The best performance was achieved by the DL model (BERT) with an average F1 score of 0.75 but only 0.54 for the PRR class. The second best-performing model was the Di-LL, with an average F1 score of 0.70 and 0.47 for the PRR class. The performance of other DL models turned out to be poorer, with the average F1 scores being in the range of 0.57–0.65. The classic SML models achieved scores that were similar to ones of the dictionary models, with the best-performing model (i.e., LR) achieving an average F1 score of 0.70 and 0.48 for the PRR class.

**Table 4:** Models’ performance on the document-based test dataset

	No preprocessing	Stopwords	Stemming	Stemming + stopword removal	Lemmatization	Lemmatization + stopword removal
Di-Gr	0.37 [0.62]	0.37 [0.62]	0.32 [0.63]	0.35 [0.65]	0.27 [0.60]	0.38 [0.66]
Di-LL	0.34 [0.62]	0.27 [0.60]	**0.47 [0.70]**	0.38 [0.67]	0.33 [0.61]	0.33 [0.60]
Di-Gr-LL	**0.43 [0.69]**	0.39 [0.66]	0.44 [0.69]	0.43 [0.67]	0.31 [0.63]	0.36 [0.62]
SML [PA]	0.41 [0.65]	0.33 [0.61]	0.31 [0.60]	0.44 [0.69]	0.41 [0.66]	0.31 [0.61]
SML [BNB]	0.33 [0.64]	0.23 [0.59]	0.27 [0.60]	0.17 [0.56]	0.32 [0.64]	0.25 [0.60]
SML [MNB]	0.24 [0.59]	0.37 [0.66]	0.16 [0.55]	0.18 [0.55]	0.19 [0.57]	0.24 [0.59]
SML [LR]	0.25 [0.6]	0.44 [0.69]	0.38 [0.67]	0.48 [0.70]	0.41 [0.66]	0.43 [0.66]
SML [SGD]	0.38 [0.61]	0.27 [0.60]	0.39 [0.65]	0.45 [0.68]	0.45 [0.68]	0.40 [0.67]
DL [CNN]	0.33 [0.60]	0.25 [0.57]	0.33 [0.60]	0.25 [0.57]	0.29 [0.60]	0.24 [0.59]
DL [LSTM]	0.32 [0.61]	0.35 [0.63]	0.32 [0.61]	0.35 [0.63]	0.32 [0.63]	0.38 [0.65]
DL [BERT]	0.43 [0.67]	**0.48 [0.72]**	0.46 [0.70]	**0.48 [0.72]**	**0.54 [0.75]**	**0.50 [0.73]**

The impact of preprocessing in the case of the document-based test dataset was also similar to the train-test split test dataset: in contrast to the sentence-based test dataset, where preprocessing had a major impact on models’ performance, here it caused less substantial variation. While in the case of the best-performing model (i.e., BERT), the difference between F1 scores for the lemmatization and the no preprocessing model was 0.11, for the majority of preprocessing modes, the variation between F1 scores was in the range of 0.04–0.06. More intensive modes of preprocessing overall showed better performance for the best-performing SML and DL models, whereas in the case of dictionaries, the impact of preprocessing was inconsistent.

Overall, the performance of PRR content detection models was suboptimal for two out of three test datasets. The DL model—i.e., BERT—showed the best performance across all three datasets, but its performance for the detection of PRR class for the train-test split test dataset and the document-based test dataset turned out to be low (i.e., F1 scores of 0.51 and 0.54). The second best-performing model was the one using a combination of log-likelihood and Gründl’s dictionary; however, its performance for the PRR class detection was even lower (i.e., F1 scores of 0.22 and 0.44). The sentence-based test dataset was the only dataset on which models showed acceptable performance, with BERT achieving F1 scores of 0.85 both on average and for the PRR class.

### Ensemble PRR content detection models

**Fig. 1:**
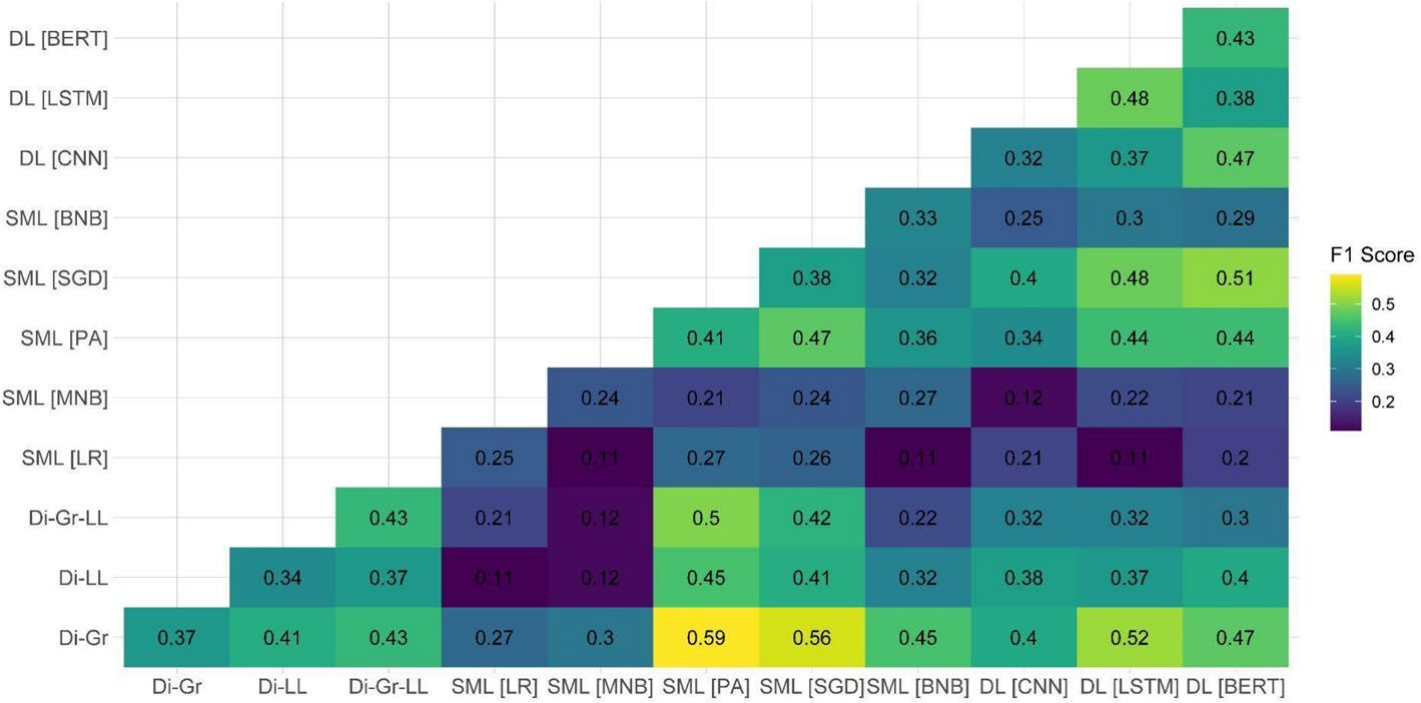
Performance of ensemble models on the document-based test dataset for no preprocessing

**Fig. 2:**
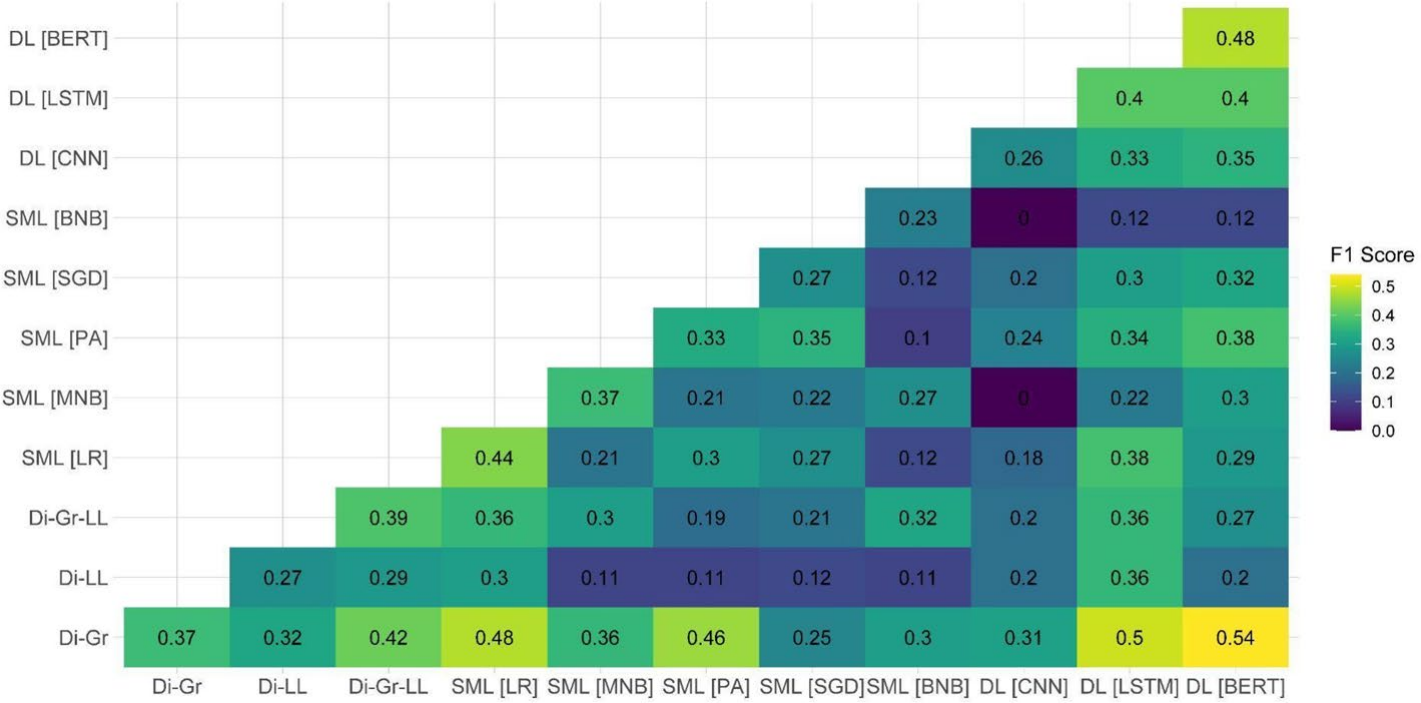
Performance of ensemble models on the document-based test dataset for stopword removal

**Fig. 3:**
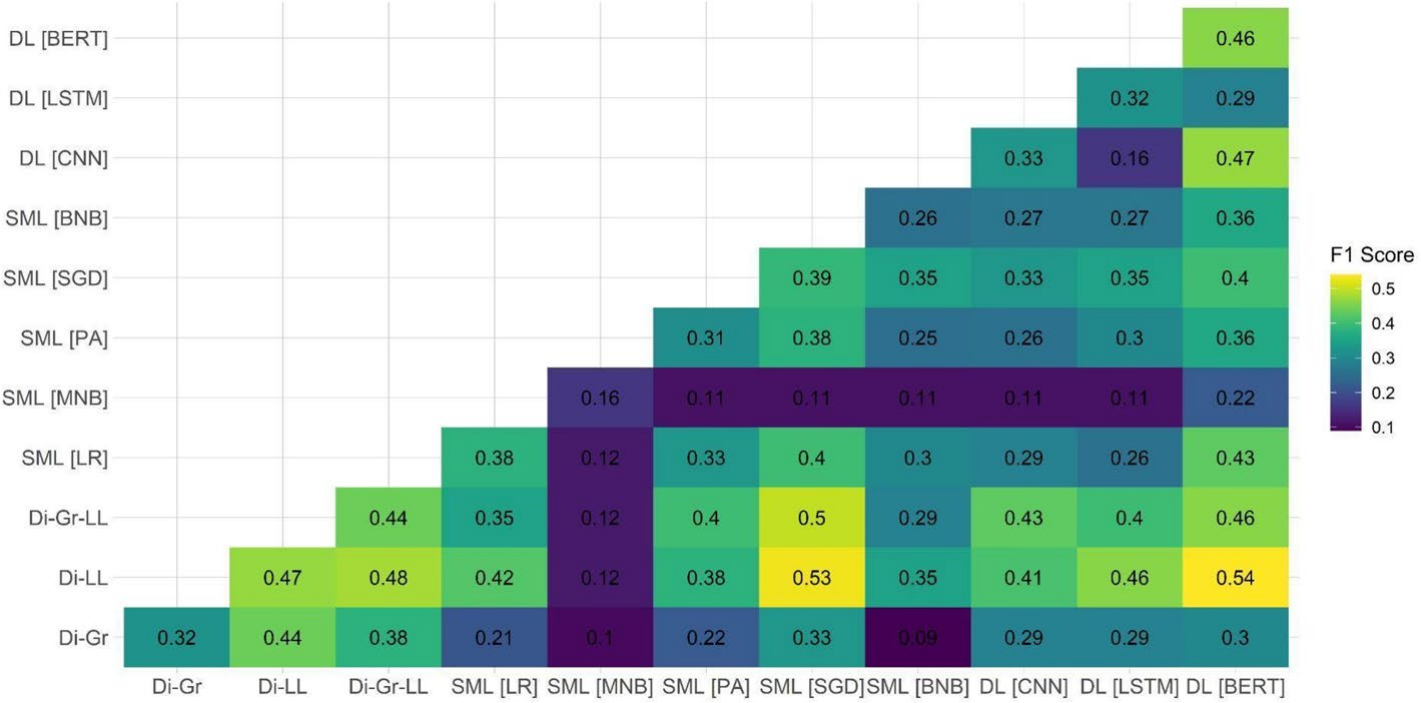
Performance of ensemble models on the document-based test dataset for stemming

**Fig. 4:**
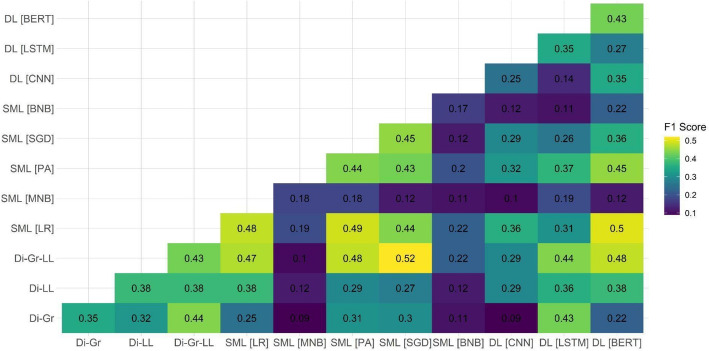
Performance of ensemble models on the document-based test dataset for stemming and stopword removal

**Fig. 5:**
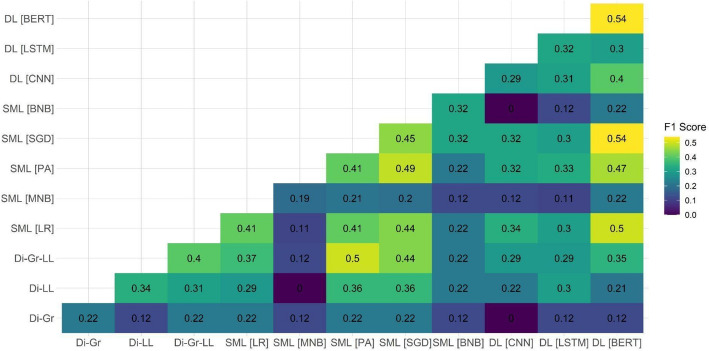
Performance of ensemble models on the document-based test dataset for lemmatization

**Fig. 6:**
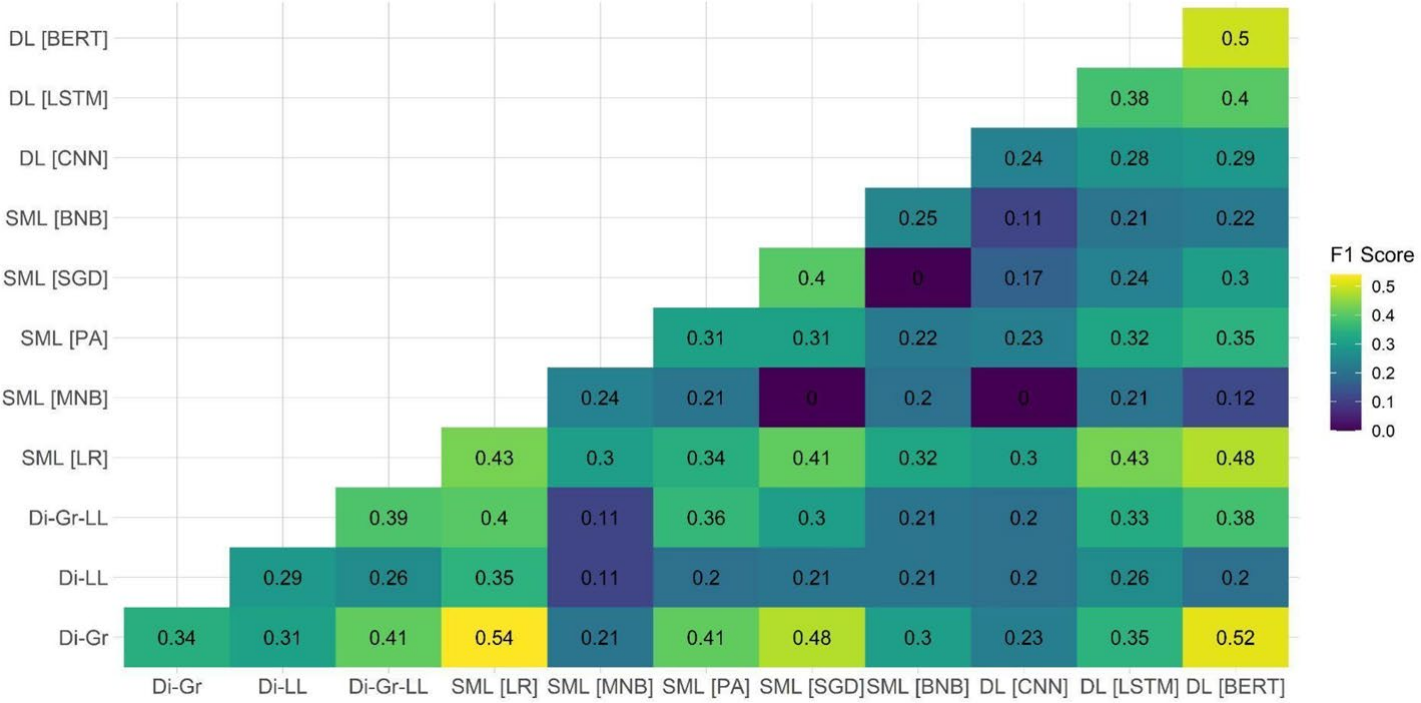
Performance of ensemble models on the document-based test dataset for lemmatization and stopword removal

After observing the suboptimal performance of individual models on two out of the three test datasets, we decided to examine the performance of ensemble models on the document-based test dataset. Our focus on this specific dataset was attributed to it being the closest to the eventual aim of the task, which is the ability to detect PRR content on the document level. Because the main shortcomings of individual models were related to the PRR class detection, Figs. 1, 2, 3, 4, 5 and 6 show F1 scores for the PRR class for each preprocessing mode (for the average and non-PRR class scores, see Appendix A7). In addition to the ensemble models’ scores, figures also show individual scores for each model (e.g., BERT and BERT cell) for easier comparison.

Figure 1 shows that in the case of the no preprocessing mode, several ensemble models provided an improvement over the baseline of F1 score of 0.43 for the PRR class achieved by individual Di-Gr-LL and BERT models. Specifically, the combination of dictionaries (Di-Gr) with the best performing SML (PA and SGD) and DL models (BERT) allowed to improve the performance up to 0.16 points (i.e., 0.59 for the Di-Gr + PA ensemble).

In the case of the stopword removal mode (Fig. 2), the improvements provided by ensembles in comparison with the baseline (i.e., 0.48 for BERT) were less substantial. As for the no preprocessing mode, the ensemble of dictionaries (Di-Gr) and DL models ( BERT and LSTM) enabled the performance increase up to 0.50 (Di-Gr + LSTM ensemble) and 0.54 (Di-Gr + BERT ensemble). Other ensemble models resulted in poorer performance, in particular in the case of the ensembles of BNB + CNN and MNB + CNN.

For the stemming mode (Fig. 3), the individual baseline of F1 score of 0.47 (Di-LL) was again improved by the combination of dictionaries with the log-likelihood-based component (Di-LL and Di-Gr-LL) with the SML (SGD) and DL (BERT) models. Similar to the stopword removal mode, the increase in performance was relatively small: the ensemble models achieved F1 scores of 0.53 (Di-LL + SGD) and 0.54 (Di-LL + BERT).

In the case of stemming with stopword removal (Fig. 4), the improvement compared with the individual model baseline (0.48 for BERT) turned out even less significant. The highest result achieved by the ensemble model (Di-Gr-LL + BERT) was only 0.04 points higher; even lesser improvements of 0.01 and 0.02 were achieved using the ensembles of SML (PA + LR) and DL models (BERT + LR).

The pattern of decreasing returns continued in the case of lemmatization (Fig. 5), where none of the ensemble models achieved performance higher than the individual model baseline (0.54 for BERT). The ensemble of BERT and SGD achieved the same performance, with the second-best ensembles of BERT + LR and PA + Di-Gr-LL achieving F1 scores of 0.5.

Finally, in the case of lemmatization with stopword removal (Fig. 6), ensemble models performed similarly to the case of stemming. While some minor improvements compared to the baseline (i.e., the F1 score of 0.5 in the case of BERT) were achieved by the combinations of Di-Gr + LR (0.54) and Di-Gr + BERT (0.52), these improvements remained relatively minor.

Altogether, the use of ensemble models shows that they are able to improve performance over individual models’ baselines for most modes of preprocessing in the case of the document-level dataset. However, only one ensemble model out of 330 managed to achieve higher performance compared with the best-performing individual option across all modes of preprocessing. The relatively limited improvement (i.e., F1 score of 0.59 as contrasted with the F1 score of 0.54 of the individual BERT model) suggests that ensemble models do not necessarily solve challenges of using computational approaches for PRR content detection; furthermore, in a number of cases, the performance of ensemble models was actually poorer than the performance of individual models.

The systematic comparison of the performance of different ensemble models also suggests that improvements are usually achieved when combining more complex SML/DL models with simpler dictionary approaches. A pattern that can be observed throughout most ensemble models suggests that the combination of distinct computational approaches allows for achieving the best results. The limited improvement of ensembles using different SML models might be due to them being trained on the same data and based on comparable algorithmic approaches. On the other hand, dictionaries rely on a different principle and, thus, might be capable of detecting PRR content that SML and DL models might have missed.

## Discussion

In this article, we examined the possibilities of using computational approaches for the automated detection of PRR content in German. Operationalizing PRR ideas as a complex construct made of three components—populism, authoritarianism, and nativism—we systematically compared the performance of 66 individual and 330 ensemble models for PRR content detection and how these models’ performance is affected by different modes of text preprocessing. Such comparison is important for the ability of scholars to detect the presence of PRR content in online environments automatically and is essential for addressing a number of research tasks related to the understanding of the spread of PRR ideas, the appearance of these ideas in the content with which individuals engage online, and the potential effects of such engagement. The importance of studying how different computational approaches perform on the PRR content detection task is amplified by the substantial risks the spread of PRR content in online environments poses for liberal democracies and the potential of automated tools capable of detecting PRR content to (at least partially) mitigate these risks. Based on this comparison, we generated several observations concerning the pitfalls and promises of the computational detection of PRR content.

Our first observation relates to the challenging nature of PRR content detection. The performance of individual models varied substantially across the three test datasets; however, on average, the DL models (i.e., BERT) demonstrated the best performance. While classic SML models rarely showed the top performance, some of them (e.g., LR) showed robust performance on the broad selection of datasets. In several cases, dictionary models outperformed both DL and classic SML models, but their performance was less consistent and more subjected to processing-based fluctuations. Such differences in performance can be attributed both to the complex nature of the task, which is more dependent on the argument structure than on the linguistic cues (hence, the lower performance of dictionaries) and to the relatively small volume of training data (in particular, concerning the actual PRR content).

In terms of individual test datasets, we observed satisfactory performance of PRR content detection models on the sentence-level test dataset, thus indicating the feasibility of computational approaches for the task. However, the performance on the other two test datasets was substantially poorer, in particular concerning the detection of items belonging to the PRR content (as contrasted by the detection of non-PRR items). While some individual models managed to reach acceptable performance on average for the document-level test dataset, such as a 0.75 F1 score for BERT or 0.70 for LR, the F1 scores for the PRR class were substantially lower. The highest score achieved was 0.54 in the case of BERT, which essentially means that the model fails to correctly classify almost half of PRR content. Such poor performance can be attributed to several reasons, including the higher noise for the document-level detection as well as the unbalanced ratio of PRR to non-PRR items in the train-test split test dataset and document-based test dataset. It also stresses the importance of using multiple test datasets for evaluating the performance of computational approaches, in particular the ones applied to complex computational tasks where the likelihood of encountering highly imbalanced and noisy data is particularly high.

The second observation concerns the substantial variation in the impact of preprocessing on models’ performance depending on the test dataset and the model used. In the case of the train-test split test dataset and the document-based test dataset (i.e., the two datasets with less balanced data composition and higher noise in the case of the document-based test dataset), the impact of preprocessing remained relatively minor, whereas in the case of the sentence-based test dataset, the difference between specific preprocessing modes turned out rather large. More computationally demanding modes, such as stemming and lemmatization, generally showed better performance for the best-performing models; among these modes, lemmatization showed more robust performance across test datasets which is in line with the common criticism of stemming as a mode of preprocessing due to the possibility of it causing data losses.

While in a few cases (e.g., BERT’s performance on the train-test split test dataset or LR’s on the sentence-based test dataset), the absence of any preprocessing enabled better performance compared to a number of the complex modes of preprocessing, in most cases preprocessing improved the performance of individual models. The most beneficial mode of preprocessing, however, varied across individual models, thus highlighting the importance of testing several preprocessing approaches when deciding on the exact implementation of the computational approach.

Our third observation relates to the potential of ensemble models, where individual detection models are combined to improve performance. While in some cases, ensemble models showed some increases in average F1 scores, these models’ performance for detecting the PRR class did not necessarily increase. Out of 330 ensemble models, only one showed better performance for the PRR class detection than the top-performing BERT-based model. It suggests that while ensemble models can increase performance for each of the individual models, such increase shall not be taken for granted. Not all models improved significantly, but most had at least one combination that helped improve performance. In this sense, the BERT model is an outlier with high individual scores, where most models, regardless of their type, benefitted from the ensemble approach.

Together, our observations can be translated into some practical recommendations for the use of computational approaches for detecting PRR content and, to a certain degree, other forms of textual content that are not necessarily defined by a small selection of linguistic cues. When the process of detection relies on a single model trained on a relatively small volume of data, DL and classic SML models seem to be the most promising approaches and can potentially demonstrate even better results with additional fine-tuning. While ensemble models might potentially achieve better performance on the noisy data, the improvement is not guaranteed. In terms of preprocessing, more computationally intensive modes, in particular lemmatization, tend to enable more consistent performance increases, albeit the exact effects of preprocessing might vary depending on the type of data to which the computational approach is applied.

These findings can be transferred to other computational tasks related to the detection of the content related to specific political phenomena. While the case of PRR content is quite distinct due to the multidimensionality and complexity of the phenomenon, our article aligns with earlier research (e.g., Ulinskaitė & Pukelis 2021; Dai & Kustov 2022) that demonstrates that DL and classic SML models have potential for identifying the complex forms of political reasoning in (online) information environments. Most earlier studies, however, tend to focus on relatively uniform types of content and the spread of PRR ideas across diverse platforms stresses the importance of looking at the ability of DL and classic SML models to deal with content with the varying amount of noise. It is what we do in our article using three test datasets with different degrees of noise, which come from the set of web-tracking data capturing user activities across different online platforms. The performance of DL and classic SML models under these circumstances suggest that these models are also likely to perform better than dictionary-based approaches for other similar tasks, for instance, regarding the detection of other types of political ideas in the textual content, especially, considering that we tested these models on sentences and documents coming from a broad range of online platforms. At the same time, our article also stresses the importance of looking for more advanced approaches for de-noising data, in particular for political content-related detection tasks, which seems to be an important prerequisite for successful models’ performance in this context.

It is important to mention the limitations of the current study and the directions for further research. The first limitation regards our focus on low-cost implementations of the detection approaches without using additional fine-tuning or training resources (e.g., increases in the number of epochs for DL-based models). Such a focus is attributed to the expectation that many academia-based projects would have limited resources for implementing such approaches, but it should be taken into consideration that a comparison of high-cost implementations might change the results. A related limitation concerns the fact that it is difficult to make the comparison of automated PRR content detection approaches exhaustive. While we compared the performance of a number of models, there are more options that can be compared, specifically, when considering SML models. While the five SML models we examined show generally solid performance and, in some cases, perform similarly to more complex DL models, it is important to note that there are other SML models that are frequently used for the detection of PRR and politics-related content. Support vector machines and random forest models are particularly relevant in this context (Grimmer & Stewart 2013), with the latter type of models showing high performance in the context of populism detection (Dai & Kustov 2022; Di Cocco & Monechi 2022). Future research will greatly benefit from drawing comparison between these models and the ones tested in this study.

Second, instead of training separate models for detecting each of the three components of PRR ideas (i.e., authoritarianism, nativism, and populism), we opted for detecting PRR content as an aggregate and fragmented construct that might not be an optional solution both conceptually and performance-wise. In particular, it is important to acknowledge the importance of non-compensatory treatment of complex multidimensional concepts such as PRR ideas (Wuttke et al., 2020). The aggregate treatment of PRR content, where the PRR label is assigned to the sentence even if it includes content aligning with a single element of PRR ideas (e.g., nativism), is, indeed, contradictory to the above-mentioned critique. However, this decision is related to the limited amount of training data for individual PRR components as well as our unit of analysis: due to our focus on sentence-level detection, it was hardly possible to find sentences containing all three PRR components. Furthermore, earlier research (e.g., Engesser et al. 2017) suggests the tendency of populism manifesting in a fragmented way in online environments, thus making it close to impossible to operationalize it in a non-compensatory way for automated detection and, potentially, justifying the use of a compensatory approach (especially, for the document-level detection which takes into consideration multiple sentences present in the document and is more likely to includes different components of PRR ideas). It is also important to note that considering our training data composition that was primarily coming from the websites of PRR media, it is unlikely that the populism-related content we detect will be related to the parts of the political spectrum other than the right one.

Nevertheless, future research can address this shortcoming in several ways. One of them regards the development of models for automated detection of content associated with individual PRR components. We have started working on this approach (also keeping in mind the shortcomings of the aggregate detection approach), but while some initial findings are promising, a substantially more systematic comparison of models’ performance is required to be able to evaluate how much improvement it provides. Another approach which we discuss briefly below, regards the shift from the sentence-level detection to the paragraph-level detection to apply the model to the chunks of text where all three PRR elements are more likely to be present.

Third, it is important to acknowledge the limitations of detecting PRR content on the sentence level. While sentence-level detection has its benefits, for instance, in terms of reducing the amount of noise and enabling a more fine-grained detection of nuanced phenomena, it has substantial limitations in the case of detecting complex constructs such as PRR ideas. Under these circumstances, the approach adopted by other studies (e.g., Ulinskaitė & Pukelis 2021; Dai & Kustov 2022), where PRR ideas are detected on the basis of lengthier segments of text (e.g., a paragraph or a set of sentences of a certain length) can help achieve better results. While in our study, we were not able to verify it systematically due to the composition of our test datasets, future research can benefit from it, especially as the models trained for the current study are to be made publicly available.

Finally, because of the large number of models compared, together with the limited computational resources, we did not use cross-validation to make the evaluations of our models’ performance more robust. For future research, it is important to integrate cross-validation into the process of performance evaluation to obtain more generalizable insights. Besides, we limited the examination of the performance of ensemble models to a combination of two individual models and looked at only one out of three test datasets, whereas it might be argued that combining three or even four models can yield even better results in terms of performance. While we encourage further research to explore this avenue, the more detailed examination of the performance of ensemble models is beyond the scope of this paper.

## Figures and Tables

**Fig. 7:**
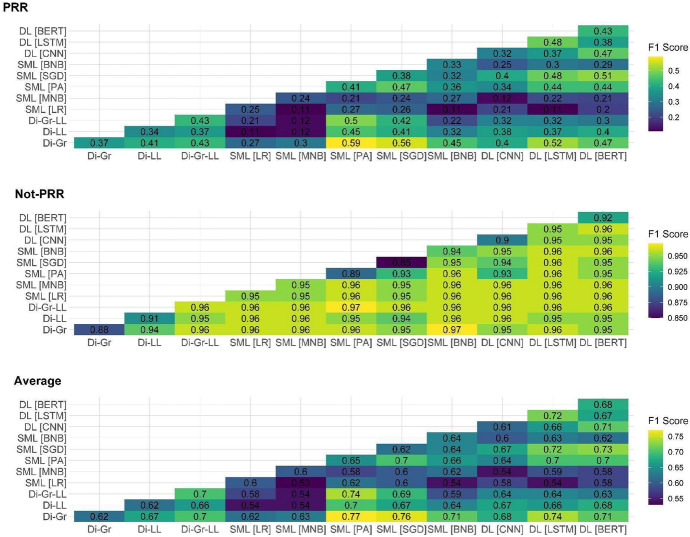
Comparison of ensemble PRR content detection models [no preprocessing]

**Fig. 8:**
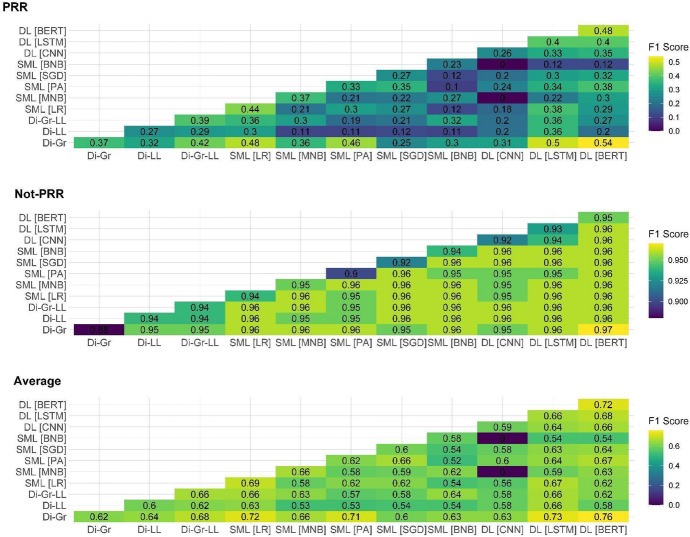
Comparison of ensemble PRR content detection models [no stopwords]

**Fig. 9:**
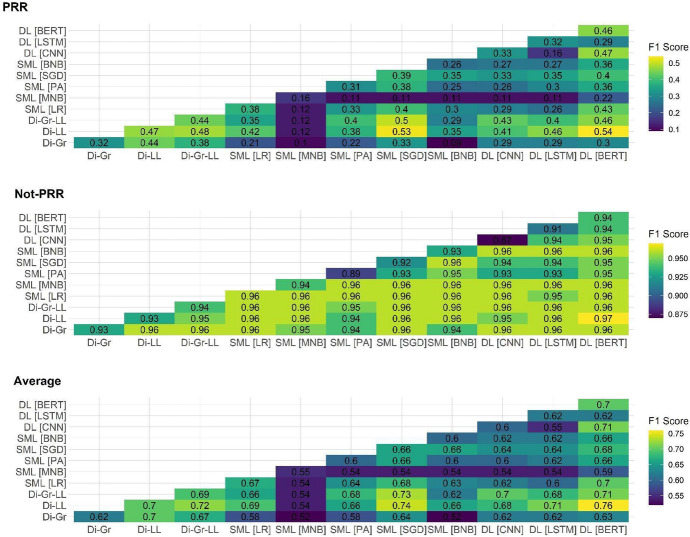
Comparison of ensemble PRR ﻿content detection models [stemming]

**Fig. 10:**
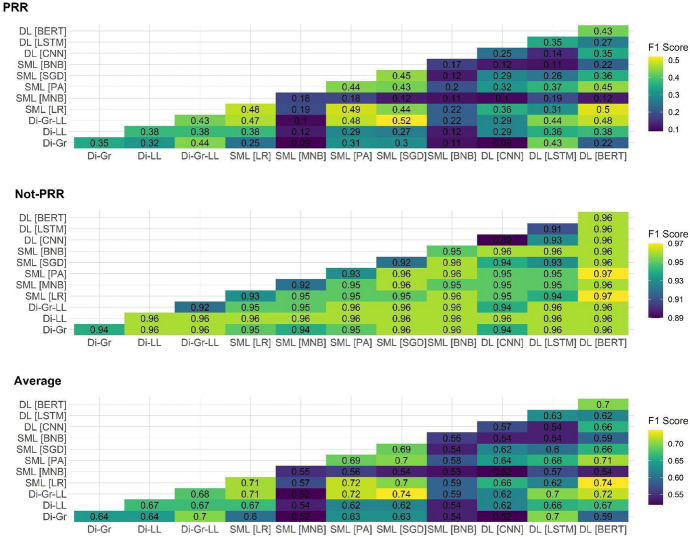
Comparison of ensemble PRR ﻿content detection models [stemming / no stopwords]

**Fig. 11:**
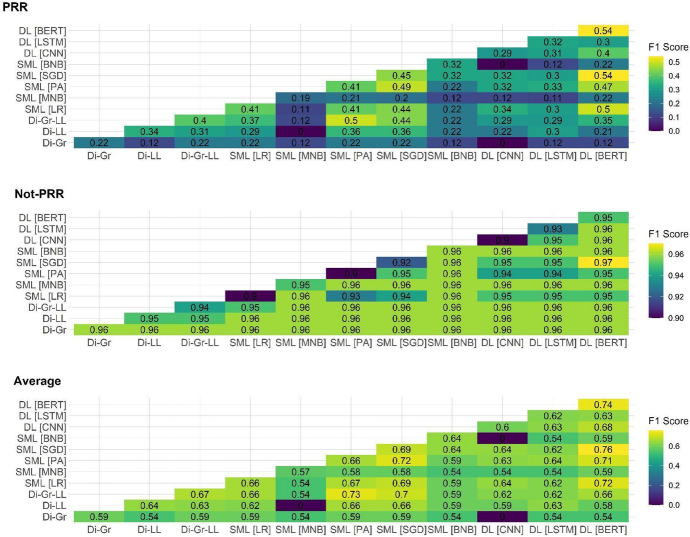
Comparison of ensemble PRR ﻿content detection models [lemmatization]

**Fig. 12:**
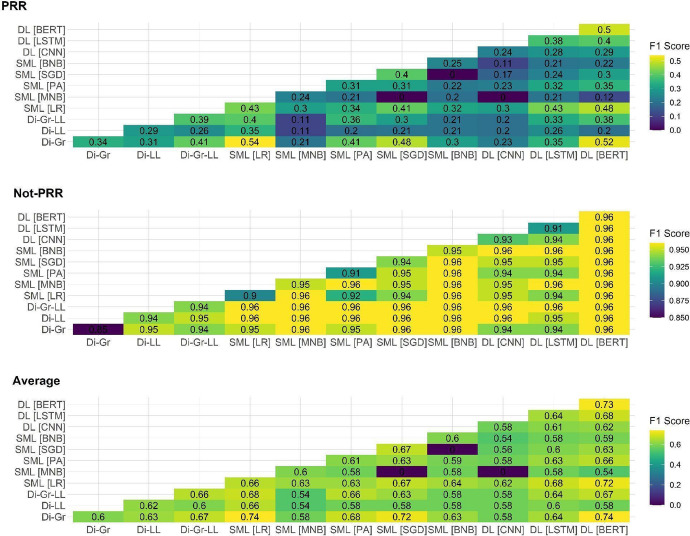
Comparison of ensemble PRR ﻿content detection models [lemmatization / no stopwords]

## Appendix

### Appendix A1: Definition of PRR content and its operationalization for manual content labeling for the training and test datasets

*Definition of PRR content*

For this paper, we follow the definition of PRR content as content dealing with a complex conceptual construct made of three key components: populism, nativism and authoritarianism (Mudde 2007). The populism component is a “thin” (Mudde 2010) anti-establishment ideology that casts the people against the elites with an emphasis on the popular will. This ideology is thin in the sense that it does not belong to a specific left- or right-wing ideology and therefore does not have a clear ideological stance on left–right cleavages such as economic policy (Mudde and Kaltwasser 2012).

The nativism component is based on the idea that only those who are native to a country are true citizens of the state (Betz 2017). This idea translates into an ethno-nationalist stance on citizenship, in which belonging is defined in exclusionist terms (i.e., as an opposition to a diverse outgroup; Betz 1994). The nativism component is often closely related to the populism component and influences how the notion of people is conceptualized; in the case of PRR, it frequently involves contrasting ethnically homogeneous citizens with migrant minorities (Abou-Chadi 2016).

The authoritarianism component emphasizes the need to conform to existing laws and rules (Inglehart and Norris 2016) and stresses the importance of security and policing (Muis and Immerzeel 2017). It encapsulates a broader desire to live in societies characterized by the order and hierarchical relations, where authority is strictly respected (Mudde 2007). In contrast to the nativism and populism components which are often treated as core components of PRR, authoritarianism is not exclusively connected to the PRR ideology and is usually associated with a broader concept of conservatism (Mudde 2010).

*Operationalization of the PRR definition for preparing training and test datasets*

The above-mentioned definition of PRR informed our approach for manual content labeling conducted to prepare a training dataset for developing PRR detection models and three test datasets (i.e., the train-test split test dataset, sentence-based test dataset and document-based test dataset) for evaluating these models’ performance. The content item (i.e., a sentence in the case of the training dataset and the train-test split test dataset/sentence-based test dataset and a document in the case of the document-based test dataset) should contain features associated with at least one of the PRR components (i.e., populism, nativism or authoritarianism) in order to be labeled as PRR.

To prepare the training and the test datasets, a team of coders manually labeled several sets of content items (for a summary of this process, see Table 5 below). In the case of the training dataset, it consisted of 27,430 unique sentences coming from 757 web pages captured in the course of the project on studying exposure of German and Swiss citizens to the PRR content (see Appendix A2 for more information). The pages were selected from the overall set of more than two million of captured Germanophone pages using structured random sampling to extract the equal share of pages coming from the right-wing hyperpartisan outlets (i.e., the ones we assumed to be likely to include PRR content; examples of such outlets include Journalistenwatch) and from non-hyperpartisan outlets containing politics-related information (e.g., journalistic media such as Bild or Facebook posts dealing with politics).

The reason to focus on non-hyperpartisan pages containing politics-related information is attributed to our assumption that PRR content is likely to be related to politics. Due to the challenging nature of the PRR content detection task, in particular in the case of web-tracking data, we decided to easen the task by first identifying politics-related content and then focusing on the computational differentiation between PRR and non-PRR content within politics-related content. To detect politics-related content, we used a dictionary-based approach made up of terms from the German codebook for the Comparative Agendas Project (CAP; Bevan 2019). The codebook is used to label political themes in Germany (e.g., economy or foreign politics) and includes key terms related to them. These terms were combined with a theoretically conceptualized list of terms for topics underrepresented in the CAP codebook (i.e., elections and ecology) and a list of political actors’ names in Germany and Switzerland (e.g., members of parliament) and G20 and EU countries (e.g., presidents and vice-ministers) to produce the final dictionary which showed acceptable performance (for more information, see Makhortykh et al. 2022).

To evaluate the performance of PRR content detection models, we used three test datasets: 1) train-test split test dataset; 2) sentence-based test dataset; and 3) document-based test dataset. The train-test split test dataset was made of 4,782 sentences (i.e., 20% of the 24,741 codable sentences in the training dataset) which were separated and not used for models’ training. The second test dataset [sentence-based test dataset] consisted of 300 sentences randomly sampled from the web pages captured in the course of the project. The selection followed the same principle as in the case of the training data: 150 sentences were sampled from the pages coming from right-wing hyperpartisan outlets and 150 sentences came from the pages originating from a broad range of platforms and containing politics-related information. Finally, the third test dataset [document-based test dataset] was made of 192 web pages randomly sampled from the sample of more than 250,000 pages captured in the course of the projects and predicted to contain politics-related information.

**Table 5:** Summary of the procedures for preparing the training and test datasets

Dataset name	Size	Source	Preparation	Coding validation
Training dataset	27,430 sentences	757 web pages from right-wing hyperpartisan (e.g., Journalistenwatch) and non-hyperpartisan (e.g.,Spiegel) outlets visited by the project participants	5 coders	After six rounds of training, the five coders coded a selected subset (N = 20) with intercoder reliability scores ranging from 0.63 to 0.78 (﻿Krippendorf’s Alpha), and inter-coder agreements ranging from 0.88 to 0.91 (Holsti coefficient)
Train-test split test dataset	4,782 sentences	Structured random sample of the sentences from the training dataset	5 coders	Same procedure as for the training dataset
Sentence-based test dataset	300 sentences	Random sample of sentences from the pages visited by the project participants	2 coders	Complete dataset checked and consensus-coded by 3 experts
Document-based test dataset	192 documents	Random sample of web pages visited by the project participants	2 coders	Complete dataset checked and consensus-coded by 3 experts

Each content item (i.e., a sentence or a document) was coded according to the following three variables: 1) NATIVISM (*i.e., Does the item contain nativism features?*); 2) AUTHORITARIANISM (*i.e., Does the item contain authoritarianism features?*); 3) POPULISM (*i.e., Does the item contain populism elements?*). A detailed description of the variables is provided below; the item was labeled as PRR if at least one of these variables was present.

Variable: NATIVISM

*Does the content item contain nativism features?*

0 = No nativism features

1 = Yes, the content item contains nativism features:

- Nativism is a form of nationalism, i.e., it is claimed that a (homogeneous) one’s own nation-state is threatened by non-native elements. These can be ideas or people, such as immigrants or people of other ethnic origins, foreign cultures or religions. It is claimed that this results in, for example, increased crime or unemployment, or that the welfare state is abused. Non-native elements are discredited, denounced or stigmatized, e.g., described as evil, criminal, lazy, bad, stupid, immoral, dangerous, etc. Non-native elements are blamed for negative, undesirable or harmful developments or situations.

Examples:

- “The desire among radical Muslims for the global domination of Islam is unbroken—and they make no secret of their plans:” [German original: “*Der Wunsch bei radikalen Muslimen nach der weltweiten Herrschaft des Islam ist ungebrochen – und sie machen kein Geheimnis aus ihren Plänen:”*].
  o *Code*: Nativism = 1
  o *Explanation*: Radical Muslims are portrayed as dangerous because they presumably strive for worldwide domination.
- “Sindelfingen, once Swabian, was the first German city where native Germans became a minority around 2018 and are dominated by 127 other nations.” [German original: “*Das einst schwäbische Sindelfingen war die erste deutsche Stadt, in der die einheimischen Deutschen um 2018 herum in die Minderheit kamen und beherrscht werden von 127 anderen Nationen.*”]
  o *Code:* Nativism = 1
  o *Explanation*: Germans are in the minority and dominated by non-native elements.
- “Immigration and the birth rate will inexorably ensure that others come to power.” [German original: “*Zuwanderung und Geburtenrate werden unaufhaltsam dafür sorgen, dass andere an die Macht kommen.*”]
  o *Code:* Nativism = 1
  o *Explanation*: Germans are threatened by non-native elements: Germans are in the minority and "the others" (here the non-nativists) are coming to power.

Variable: AUTHORITARIANISM

*Does the content item contain authoritarianism features?*

0 = No authoritarian elements

1 = Yes, the *content item* contains *authoritarianism features.*

Authoritarianism* features are*:

- A strong political leadership
- Emphasis on law and order/a strictly ordered society (the emphasis here is on strictness, possibly even excessive harshness, in enforcing social rules (see example 1). The fact that the government issues laws does not make it an authoritarian state).
- The desire for social conformity
- Rejection of characteristics of liberal democracies, such as pluralism, minority rights, or individual’s autonomy (but at the system level, also abolition of the democratic system)
- Aggression toward “outsiders”, “other” social groups, e.g., minorities Note: aggression towards foreigners, immigrants, etc. is not coded here, but in the variable NATIVISM (but there are also other social minorities besides foreigners, these are meant here).

Examples:

- “They already control and restrict so much, what’s next?” [German original: “*Sie kontrollieren und schränken schon so viel ein, was kommt als nächstes?*”].
  o *Code*: Authoritarianism = 1
- “Japan extended current emergency regulations to the entire country over the weekend.” [German original: “*Japan hat am Wochenende die geltenden Notstandsregelungen auf das ganze Land ausgeweitet.*”]
  o *Code*: Authoritarianism = 0 (Enacting laws and regulation is the job of governments)
- “Feminism masquerades as a movement for women's rights.” [German original: “*Der Feminismus maskiert sich als Bewegung für die Frauenrechte*.”]
  o *Code*: Authoritarianism = 1
  o *Explanation*: The item is directed against feminism and expresses aggression towards women who hold this position.
- “Politicians must do what the masses cannot do themselves, develop a political program and have the leadership skills to implement it consistently.” [German original: “*Politiker müssen das machen, was die Masse selber nicht tun kann, ein politisches Programm entwickeln und die entsprechenden Führungsqualitäten haben um sie konsequent umzusetzen.*”]
  o *Code:* Authoritarianism = 1
  o *Explanation*: The item expresses desire for politicians to be strong leaders.

Variable: POPULISM

*Does the content item contain populism features?*

0 = No populism features.

1 = Yes, the content item contains populism features.

The content item contains populism features, if:

- The good citizens are contrasted with an “evil” elite: Citizens are ascribed positive qualities, e.g., moral behavior, charisma, credibility, diligence, etc.; the elite is portrayed as e.g., corrupt, malicious, criminal, lazy, stupid, evil, undemocratic, etc.
- A preference is expressed for more sovereignty of the people: The author advocates giving more power to the people (e.g., by introducing direct democratic elements or increasing political participation). The lack of popular sovereignty is criticized (e.g., the powerlessness of the people is emphasized).This demand can also refer to specific issues (e.g., elections, decisions on immigration, etc.).
- The belief in the homogeneity and virtuousness of the people is expressed: The citizens/people share a common understanding of the world, common feelings, desires, opinions, or a common will, and the citizens/people are described as positive.

Examples:

- “The world economy is collapsing, millions have lost their jobs and have no income, …. but the most important issue for the politicians and media is BLM, the hysterical search in everything for alleged racism, and other made-up bullshit, a complete distraction from what is really at stake.” [German original: “*Die Weltwirtschaft ist am zusammenbrechen, Millionen haben ihren Job verloren und kein Einkommen, … aber das wichtigste Thema für die Politiker und Medien ist BLM, die hysterische Suche in allem nach vermeindlichem Rassismus, und anderer erfundener Bullshit, eine völlige Ablenkung um was es wirklich geht.*”].
  o *Code:* Populism = 1
  o *Explanation:* “The politicians” who make mistakes and inflict harm and suffering are contrasted with the millions of the unemployed.
- “Now the taxpayers are to bear the burden from the damage done by the traitors.” [German original: “*Jetzt sollen die Steuerzahler die Last aus dem Schaden, den die Verräter angerichtet haben, tragen.*”]
  o *Code:* Populism = 1
  o *Explanation:* “The traitors” as the elite are contrasted with the taxpayers.
- “Are we as a people completely powerless?” [German original: “*Sind wir als Volk komplett machtlos?*”]
  o *Code:* Populism = 1
  o *Explanation:* The sentence questions the popular sovereignty.

### Appendix A2: Use case

Our interest in the computational approaches for PRR content detection in German is related to the specific requirements of our use case that is based on the project focused on studying exposure of German and Swiss citizens to the PRR content in online environments. To achieve its aim, the project relied on web-tracking data that allowed capturing web pages visited by the project participants on their desktop devices. Similar to other web-tracking studies (e.g., Stier et al. 2020, Merten et al. 2022), we recruited a sample of participants (N = 1,149) from Germany and Switzerland via a market research company and asked them to install web-tracking software for Chrome or Firefox browsers on their desktop devices. During the two web-tracking rounds in spring and autumn 2020, the software extracted the HTML content appearing in the browsers following the screen-scraping principle (for more information, see Christner et al. 2022). and then transferred it to the remote server.

To protect the participants’ privacy, we used a denylist (i.e., a list of websites where visits were not captured, which in this case included healthcare-related websites, online banking portals, commercial websites, and pornography websites), as well as customised filters for social media platforms (i.e., to avoid capturing personally identifiable data as well as non-public data). The remaining visits were all captured, resulting in over two million HTML pages in German language associated with approximately 80,000 unique domains.

The large volume of available data prompted our interest in computational approaches for PRR content detection. At the same time, the implementation of these approaches was complicated by the high variety of HTML pages present in the web-tracking data that made it infeasible to develop platform-specific detection solutions capable of accounting for the distinct format and style of textual data associated with a specific domain. The diversity of content also increased the volume of possible noise in the data (e.g., due to the reliance on universal and not platform-specific HTML parsers). Hence, to be able to detect PRR content in these multi-platform data, as well as to realize possibilities provided by these data (e.g., by looking at how the long tail of information consumption affects individual engagements with PRR ideas online), we needed a cross-platform means of automated detection of PRR content.

### Appendix A3: DL models’ architectures

To train the CNN- and LSTM-based models, we used tokenizers with a vocabulary size of up to 120,000 tokens. We used a batch size of 256 with five training epochs for each model, with a binary cross-entropy function for measuring the loss during the model training, and a Nadam optimiser. For both the CNN and LSTM models, we used sequential architectures with the composition of layers described in Tables 6 and 7.

**Table 6:** Composition of layers for the CNN network

Layer	Activation
Embedding	None
1D convolution layer	ReLU
Global max pooling for 1D data	None
Dropout layer	None
Dense layer	ReLU
Dropout layer	None
Dense layer	Sigmoid

**Table 7:** Composition of layers for the LSTM network

Layer	Activation
Embedding	None
Bidirectional LSTM	None
Global max pooling for 1D data	None
Dropout layer	None
Dense layer	ReLU
Dropout layer	None
Dense layer	Sigmoid

### Appendix A4: Thresholds for dictionary models

For the three dictionary models which we used for PRR content detection, the decision whether the sentence is related to PRR ideas was based on the number of unique PRR-related terms present in the sentence. We used the absolute number of unique PRR-related terms instead of the ratio of the PRR-related terms to all terms in the sentence, because we expected the former to be a more robust measure. Table 8 shows the minimal number of terms from the individual dictionary for the sentence to be labeled as the one belonging to PRR content. The thresholds are reported separately for different preprocessing modes: for instance, in the case of the Di-LL dictionary with no preprocessing mode, at least 2 terms from the dictionary shall be present in the sentence in order for it to be labeled as the PRR content item.

**Table 8:** The minimal number of PRR-related terms per sentence for each dictionary / preprocessing mode combination

Preprocessing/ dictionary	No preproc	Stopword removal	Stem	Stem + stopword	Lemma	Lemma + stopword
Di-Gr	1	1	3	3	2	2
Di-LL	2	1	2	1	2	1
Di-Gr-LL	2	1	3	2	2	2

### Appendix A5: Thresholds for document-level PRR content detection

To implement the transition between sentence-level and document-level detection, we had to identify the minimal number of sentences containing PRR-related content for the document to be labeled as the one related to PRR ideas. Because we expected that some documents may be rather long, we opted for using the ratio of sentences labeled as the ones containing PRR content to all sentences in the document instead of the absolute number of sentences with PRR content. Table 9 summarizes the minimal proportion of PRR-related sentences per document for different models per each preprocessing mode for the document to be assigned a PRR label.

**Table 9:** Optimal ratio of PRR sentences per document for detection models (per preprocessing mode)

Model	No preproc. (%)	Stopword removal (%)	Stem (%)	Stem + stopword (%)	Lemma (%)	Lemma + stopword (%)
Di-Gr	21.95	21.95	6.57	6.57	10.25	3.94
Di-LL	9.23	41.81	23.63	51.72	14.45	17.22
Di-Gr-LL	21.73	52.43	23.07	31	38.46	4.62
SML [PA]	2.5	1.6	2.4	2.4	2.6	2
SML [BNB]	2	3.4	2.2	4	3.6	3.4
SML [MNB]	3	3.4	3.4	2.4	3.4	3.4
SML [LR]	13	4.6	13.7	5.5	6.6	3.4
SML [SGD]	5.2	7.9	9.2	1.8	6.8	4.2
DL [CNN]	0.5	0.5	0.6	0.6	0.5	0.5
DL [LSTM]	1.3	0.7	2.8	1.3	2.4	0.5
DL [BERT]	0.6	1.9	16.6	5.2	1.1	5

### Appendix A6: Performance metrics for individual PRR content detection models

This appendix provides a detailed overview of the performance metrics for the individual PRR content detection models. Specifically, it provides information about the model performance for the detection of non-PRR and PRR classes together with the average performance values. Three metrics are reported: (1) *precision* (pr) defining how many of the cases identified by the model as positive were actually positive and calculated by dividing the number of true positives by the sum of true positives and false negatives; (2) *recall* (rec) defining how many of all the positive cases available were correctly identified by the model and calculated by dividing the true positive rate by the sum of true positives and false positives; and (3) F1 score (F1) providing the harmonic mean of precision and recall.

The metrics are provided in Tables 10, 11, 12, 13, 14 and 15 Each table corresponds to one of the six modes of preprocessing (e.g., no preprocessing or stemming only) and contains information about the performance of the models across the following three test datasets: (1) *train-test split test dataset* made up of a subsample of training data (4,782 sentences); 2) *sentence-based test dataset* composed of 300 sentences from right-wing hyperpartisan outlets and a broad range of other platforms; 3) *document-based test dataset* made of 192 web pages containing politics-related information.

**Table 10:** Comparison of individual PRR content detection models [no preprocessing]

	Train-test split test dataset			Sentence-based test dataset			Document-based test dataset		
	Pr	Rec	F1	Pr	Rec	F1	Pr	Rec	F1
*Di-Gr [av]*	0.52	0.56	0.49	0.67	0.65	0.65	0.61	0.75	0.62
Non-PRR	0.93	0.71	0.80	0.67	0.80	0.73	0.97	0.81	0.88
PRR	0.11	0.41	0.17	0.67	0.51	0.58	0.25	0.69	0.37
*Di-LL [av]*	0.55	0.62	0.55	0.64	0.89	0.74	0.60	0.68	0.62
Non-PRR	0.94	0.81	0.87	0.72	0.37	0.49	0.95	0.87	0.91
PRR	0.16	0.42	0.23	0.68	0.63	0.62	0.26	0.50	0.34
*Di-Gr-LL [av]*	0.54	0.62	0.51	0.72	0.70	0.70	0.72	0.67	0.69
Non-PRR	0.94	0.71	0.81	0.71	0.84	0.77	0.94	0.97	0.96
PRR	0.14	0.52	0.22	0.74	0.57	0.64	0.50	0.38	0.43
*SML [PA] [av]*	0.7	0.66	0.68	0.78	0.68	0.66	0.63	0.79	0.65
Non-PRR	0.95	0.96	0.95	0.66	0.96	0.78	0.97	0.82	0.89
PRR	0.45	0.37	0.41	0.9	0.39	0.54	0.28	0.75	0.41
*SML [BNB] [av]*	0.74	0.63	0.66	0.73	0.6	0.55	0.65	0.63	0.64
Non-PRR	0.94	0.98	0.96	0.61	0.97	0.75	0.94	0.95	0.94
PRR	0.53	0.28	0.37	0.86	0.22	0.36	0.36	0.31	0.33
*SML [MNB] [av]*	0.79	0.61	0.65	0.8	0.57	0.5	0.63	0.58	0.59
Non-PRR	0.94	0.99	0.96	0.59	1.0	0.74	0.93	0.97	0.95
PRR	0.65	0.23	0.34	1.0	0.14	0.25	0.33	0.19	0.24
*SML [LR] [av]*	0.64	0.71	0.67	0.8	0.76	0.77	0.65	0.58	0.6
Non-PRR	0.96	0.91	0.93	0.74	0.93	0.82	0.93	0.97	0.95
PRR	0.33	0.52	0.4	0.87	0.6	0.71	0.38	0.19	0.25
*SML [SGD] [av]*	0.63	0.69	0.65	0.8	0.75	0.75	0.61	0.81	0.61
Non-PRR	0.95	0.91	0.93	0.72	0.94	0.82	0.99	0.75	0.85
PRR	0.31	0.48	0.38	0.88	0.55	0.68	0.24	0.88	0.38
*DL [CNN] [av]*	0.78	0.62	0.66	0.79	0.62	0.57	0.59	0.68	0.61
Non-PRR	0.94	0.99	0.96	0.62	0.99	0.76	0.95	0.85	0.90
PRR	0.63	0.26	0.37	0.97	0.24	0.38	0.24	0.50	0.32
*DL [LSTM] [av]*	0.81	0.63	0.67	0.81	0.63	0.59	0.71	0.72	0.72
Non-PRR	0.94	0.99	0.96	0.63	1.00	0.77	0.95	0.95	0.95
PRR	0.68	0.27	0.38	1.00	0.26	0.41	0.47	0.50	0.48
*DL [BERT] [av]*	0.74	0.70	0.72	0.80	0.68	0.66	0.64	0.75	0.67
Non-PRR	0.95	0.97	0.96	0.65	0.99	0.78	0.96	0.88	0.92
PRR	0.54	0.43	0.48	0.96	0.37	0.53	0.32	0.62	0.43

**Table 11:** Comparison of individual PRR content detection models [no stopwords]

	Train-test split test dataset			Sentence-based test dataset			Document-based test dataset		
	Pr	Rec	F1	Pr	Rec	F1	Pr	Rec	F1
*Di-Gr [av]*	0.52	0.56	0.49	0.74	0.64	0.62	0.61	0.75	0.62
Non-PRR	0.93	0.71	0.80	0.64	0.95	0.77	0.97	0.81	0.88
PRR	0.11	0.41	0.17	0.85	0.34	0.48	0.25	0.69	0.37
*Di-LL [av]*	0.53	0.61	0.43	0.62	0.58	0.55	0.61	0.60	0.60
Non-PRR	0.95	0.51	0.66	0.60	0.88	0.71	0.93	0.94	0.94
PRR	0.11	0.72	0.20	0.65	0.28	0.39	0.29	0.25	0.27
*Di-Gr-LL [av]*	0.53	0.60	0.37	0.74	0.64	0.62	0.65	0.68	0.66
Non-PRR	0.96	0.39	0.56	0.64	0.95	0.77	0.95	0.93	0.94
PRR	0.11	0.81	0.19	0.85	0.34	0.48	0.35	0.44	0.39
*SML [PA] [av]*	0.73	0.65	0.68	0.8	0.64	0.6	0.60	0.68	0.61
Non-PRR	0.94	0.97	0.96	0.63	0.99	0.77	0.95	0.86	0.90
PRR	0.52	0.32	0.4	0.97	0.28	0.43	0.24	0.50	0.33
*SML [BNB] [av]*	0.75	0.64	0.67	0.74	0.59	0.54	0.61	0.57	0.59
Non-PRR	0.94	0.98	0.96	0.6	0.98	0.74	0.93	0.96	0.94
PRR	0.55	0.3	0.39	0.87	0.2	0.33	0.30	0.19	0.23
*SML [MNB] [av]*	0.78	0.61	0.65	0.8	0.58	0.51	0.70	0.64	0.66
Non-PRR	0.94	0.99	0.96	0.59	1.0	0.75	0.94	0.97	0.95
PRR	0.63	0.24	0.35	1.0	0.16	0.27	0.45	0.31	0.37
*SML [LR] [av]*	0.68	0.72	0.7	0.82	0.73	0.73	0.68	0.72	0.69
Non-PRR	0.95	0.94	0.95	0.7	0.98	0.82	0.95	0.93	0.94
PRR	0.41	0.49	0.45	0.94	0.49	0.65	0.40	0.50	0.44
*SML [SGD] [av]*	0.54	0.63	0.39	0.74	0.69	0.65	0.59	0.61	0.60
Non-PRR	0.97	0.4	0.57	0.9	0.43	0.59	0.94	0.91	0.91
PRR	0.11	0.86	0.2	0.57	0.94	0.71	0.24	0.31	0.27
*DL [CNN] [av]*	0.81	0.63	0.67	0.74	0.59	0.54	0.58	0.61	0.59
Non-PRR	0.94	0.99	0.96	0.60	0.98	0.75	0.94	0.90	0.92
PRR	0.68	0.27	0.38	0.88	0.21	0.34	0.23	0.31	0.26
*DL [LSTM] [av]*	0.75	0.64	0.68	0.78	0.62	0.58	0.64	0.70	0.67
Non-PRR	0.94	0.98	0.96	0.62	0.99	0.76	0.95	0.91	0.93
PRR	0.56	0.30	0.39	0.94	0.25	0.40	0.33	0.50	0.40
*DL [BERT] [av]*	0.71	0.73	0.72	0.77	0.72	0.71	0.71	0.72	0.72
Non-PRR	0.95	0.95	0.95	0.66	0.94	0.78	0.95	0.95	0.95
PRR	0.47	0.51	0.49	0.88	0.51	0.64	0.47	0.50	0.48

**Table 12:** Comparison of individual PRR content detection models [stemming]

	Train-test split test dataset			Sentence-based test dataset			Document-based test dataset		
	Pr	Rec	F1	Pr	Rec	F1	Pr	Rec	F1
*Di-Gr [av]*	0.53	0.53	0.53	0.71	0.59	0.54	0.61	0.64	0.63
Non-PRR	0.92	0.93	0.93	0.60	0.96	0.74	0.94	0.91	0.93
PRR	0.14	0.14	0.14	0.82	0.21	0.33	0.29	0.38	0.32
*Di-LL [av]*	0.54	0.62	0.47	0.70	0.70	0.70	0.67	0.76	0.70
Non-PRR	0.95	0.59	0.73	0.74	0.71	0.73	0.96	0.90	0.93
PRR	0.12	0.64	0.20	0.66	0.69	0.68	0.37	0.62	0.47
*Di-Gr-LL [av]*	0.53	0.60	0.49	0.71	0.71	0.71	0.68	0.72	0.69
Non-PRR	0.94	0.66	0.78	0.73	0.77	0.75	0.95	0.93	0.94
PRR	0.12	0.55	0.20	0.69	0.65	0.67	0.40	0.50	0.44
*SML [PA] [av]*	0.72	0.66	0.68	0.79	0.66	0.64	0.59	0.67	0.60
Non-PRR	0.94	0.97	0.96	0.65	0.98	0.78	0.95	0.84	0.89
PRR	0.49	0.35	0.41	0.94	0.34	0.49	0.22	0.50	0.31
*SML [BNB] [av]*	0.71	0.64	0.66	0.73	0.61	0.57	0.60	0.59	0.60
Non-PRR	0.94	0.97	0.96	0.62	0.96	0.75	0.93	0.94	0.93
PRR	0.47	0.3	0.37	0.85	0.25	0.39	0.27	0.25	0.26
*SML [MNB] [av]*	0.79	0.6	0.64	0.79	0.55	0.45	0.57	0.54	0.55
Non-PRR	0.93	0.99	0.96	0.58	1.0	0.73	0.92	0.96	0.94
PRR	0.64	0.21	0.32	1.0	0.1	0.18	0.22	0.12	0.16
*SML [LR] [av]*	0.64	0.72	0.67	0.78	0.74	0.74	0.72	0.64	0.67
Non-PRR	0.96	0.9	0.93	0.73	0.91	0.81	0.94	0.97	0.96
PRR	0.32	0.55	0.41	0.84	0.57	0.68	0.50	0.31	0.38
*SML [SGD] [av]*	0.62	0.7	0.64	0.79	0.75	0.75	0.63	0.72	0.65
Non-PRR	0.95	0.89	0.92	0.73	0.92	0.81	0.96	0.88	0.92
PRR	0.29	0.51	0.37	0.85	0.57	0.68	0.30	0.56	0.39
*DL [CNN] [av]*	0.77	0.64	0.68	0.76	0.60	0.55	0.59	0.71	0.60
Non-PRR	0.94	0.98	0.96	0.61	0.98	0.75	0.96	0.80	0.87
PRR	0.61	0.30	0.40	0.91	0.22	0.35	0.22	0.62	0.33
*DL [LSTM] [av]*	0.73	0.66	0.69	0.74	0.64	0.62	0.60	0.66	0.61
Non-PRR	0.94	0.97	0.96	0.64	0.95	0.76	0.95	0.88	0.91
PRR	0.52	0.35	0.42	0.83	0.34	0.48	0.25	0.44	0.32
*DL [BERT] [av]*	0.61	0.77	0.62	0.85	0.85	0.85	0.67	0.74	0.70
Non-PRR	0.97	0.78	0.87	0.87	0.85	0.86	0.96	0.92	0.94
PRR	0.24	0.76	0.37	0.83	0.86	0.85	0.39	0.56	0.46

**Table 13:** Comparison of individual PRR content detection models [stemming / no stopwords]

	Train-test split test dataset			Sentence-based test dataset			Document-based test dataset		
	Pr	Rec	F1	Pr	Rec	F1	Pr	Rec	F1
*Di-Gr [av]*	0.51	0.54	0.41	0.63	0.64	0.63	0.64	0.65	0.65
Non-PRR	0.93	0.52	0.67	0.69	0.61	0.65	0.94	0.93	0.94
PRR	0.09	0.57	0.16	0.58	0.66	0.62	0.33	0.38	0.35
*Di-LL [av]*	0.54	0.63	0.40	0.74	0.73	0.72	0.72	0.64	0.67
Non-PRR	0.97	0.43	0.59	0.84	0.61	0.71	0.94	0.97	0.96
PRR	0.11	0.82	0.20	0.64	0.86	0.73	0.50	0.31	0.38
*Di-Gr-LL [av]*	0.54	0.62	0.46	0.72	0.72	0.72	0.64	0.75	0.67
Non-PRR	0.95	0.57	0.72	0.78	0.69	0.73	0.96	0.88	0.92
PRR	0.12	0.67	0.21	0.66	0.75	0.70	0.32	0.62	0.43
*SML [PA] [av]*	0.74	0.66	0.69	0.8	0.64	0.6	0.65	0.76	0.69
Non-PRR	0.94	0.97	0.96	0.63	0.99	0.77	0.96	0.89	0.93
PRR	0.53	0.34	0.42	0.97	0.28	0.43	0.34	0.62	0.44
*SML [BNB] [av]*	0.73	0.65	0.67	0.75	0.61	0.56	0.61	0.55	0.56
Non-PRR	0.94	0.97	0.96	0.61	0.98	0.75	0.92	0.97	0.95
PRR	0.51	0.32	0.39	0.89	0.24	0.38	0.29	0.12	0.17
*SML [MNB] [av]*	0.77	0.61	0.64	0.79	0.56	0.48	0.55	0.55	0.55
Non-PRR	0.94	0.99	0.96	0.58	1.0	0.74	0.93	0.91	0.92
PRR	0.6	0.22	0.33	1.0	0.12	0.21	0.17	0.19	0.18
*SML [LR] [av]*	0.66	0.71	0.68	0.81	0.74	0.74	0.67	0.79	0.70
Non-PRR	0.95	0.92	0.94	0.72	0.96	0.82	0.97	0.89	0.93
PRR	0.37	0.51	0.42	0.91	0.53	0.67	0.37	0.69	0.48
*SML [SGD] [av]*	0.66	0.65	0.65	0.79	0.67	0.65	0.65	0.78	0.68
Non-PRR	0.94	0.95	0.95	0.66	0.98	0.78	0.97	0.88	0.92
PRR	0.39	0.34	0.36	0.92	0.37	0.52	0.33	0.69	0.45
*DL [CNN] [av]*	0.77	0.63	0.67	0.79	0.61	0.56	0.56	0.61	0.57
Non-PRR	0.94	0.98	0.96	0.61	0.99	0.76	0.94	0.85	0.89
PRR	0.59	0.28	0.38	0.97	0.22	0.35	0.19	0.38	0.25
*DL [LSTM] [av]*	0.74	0.66	0.69	0.77	0.64	0.61	0.61	0.69	0.63
Non-PRR	0.94	0.97	0.96	0.64	0.98	0.77	0.95	0.88	0.91
PRR	0.54	0.34	0.42	0.91	0.31	0.46	0.27	0.50	0.35
*DL [BERT] [av]*	0.67	0.73	0.69	0.79	0.77	0.77	0.72	0.67	0.69
Non-PRR	0.95	0.92	0.93	0.71	0.89	0.79	0.94	0.97	0.96
PRR	0.38	0.54	0.45	0.87	0.65	0.75	0.50	0.38	0.43

**Table 14:** Comparison of individual PRR content detection models [lemmatization]

	Train-test split test dataset			Sentence-based test dataset			Document-based test dataset		
	Pr	Rec	F1	Pr	Rec	F1	Pr	Rec	F1
*Di-Gr [av]*	0.52	0.54	0.52	0.64	0.58	0.55	0.61	0.60	0.60
Non-PRR	0.93	0.86	0.89	0.60	0.90	0.72	0.93	0.94	0.94
PRR	0.12	0.21	0.16	0.68	0.27	0.39	0.29	0.25	0.27
*Di-LL [av]*	0.54	0.62	0.51	0.73	0.72	0.72	0.59	0.70	0.61
Non-PRR	0.95	0.69	0.80	0.73	0.83	0.77	0.95	0.83	0.89
PRR	0.13	0.55	0.22	0.74	0.62	0.67	0.23	0.56	0.33
*Di-Gr-LL [av]*	0.53	0.61	0.45	0.75	0.75	0.75	0.67	0.61	0.63
Non-PRR	0.95	0.55	0.70	0.82	0.69	0.75	0.93	0.97	0.95
PRR	0.12	0.67	0.20	0.68	0.81	0.74	0.40	0.25	0.31
*SML [PA] [av]*	0.73	0.66	0.69	0.8	0.65	0.62	0.63	0.77	0.66
Non-PRR	0.94	0.97	0.96	0.64	0.99	0.78	0.97	0.85	0.90
PRR	0.51	0.35	0.42	0.95	0.31	0.47	0.29	0.69	0.41
*SML [BNB] [av]*	0.73	0.63	0.67	0.74	0.61	0.57	0.97	0.59	0.64
Non-PRR	0.94	0.98	0.96	0.61	0.97	0.75	0.93	1.00	0.96
PRR	0.52	0.29	0.38	0.87	0.25	0.38	1.00	0.19	0.32
*SML [MNB] [av]*	0.79	0.61	0.65	0.76	0.56	0.48	0.66	0.55	0.57
Non-PRR	0.94	0.99	0.96	0.59	0.99	0.74	0.93	0.98	0.95
PRR	0.65	0.23	0.34	0.94	0.13	0.22	0.40	0.12	0.19
*SML [LR] [av]*	0.64	0.72	0.67	0.82	0.78	0.78	0.63	0.77	0.66
Non-PRR	0.96	0.9	0.93	0.75	0.95	0.84	0.97	0.85	0.90
PRR	0.32	0.53	0.4	0.9	0.6	0.72	0.29	0.69	0.41
*SML [SGD] [av]*	0.65	0.69	0.66	0.8	0.73	0.73	0.65	0.78	0.68
Non-PRR	0.95	0.92	0.94	0.7	0.96	0.81	0.97	0.88	0.92
PRR	0.34	0.45	0.39	0.91	0.5	0.64	0.33	0.69	0.45
*DL [CNN] [av]*	0.81	0.63	0.67	0.79	0.60	0.54	0.58	0.65	0.60
Non-PRR	0.94	0.99	0.96	0.61	0.99	0.75	0.94	0.86	0.90
PRR	0.69	0.27	0.38	0.96	0.20	0.33	0.22	0.44	0.29
*DL [LSTM] [av]*	0.75	0.65	0.68	0.80	0.64	0.61	0.61	0.64	0.63
Non-PRR	0.94	0.98	0.96	0.63	0.99	0.77	0.94	0.91	0.93
PRR	0.56	0.32	0.41	0.98	0.29	0.45	0.29	0.38	0.32
*DL [BERT] [av]*	0.73	0.73	0.73	0.80	0.74	0.73	0.72	0.78	0.75
Non-PRR	0.96	0.95	0.96	0.70	0.96	0.81	0.96	0.94	0.95
PRR	0.50	0.51	0.51	0.91	0.51	0.66	0.48	0.62	0.54

**Table 15:** Comparison of individual PRR content detection models [lemmatization / no stopwords]

	Train-test split test dataset			Sentence-based test dataset			Document-based test dataset		
	Pr	Rec	F1	Pr	Rec	F1	Pr	Rec	F1
*Di-Gr [av]*	0.52	0.54	0.53	0.64	0.58	0.55	0.64	0.68	0.66
Non-PRR	0.93	0.87	0.90	0.60	0.90	0.72	0.95	0.92	0.93
PRR	0.12	0.20	0.15	0.68	0.27	0.39	0.33	0.44	0.38
*Di-LL [av]*	0.54	0.63	0.41	0.74	0.74	0.72	0.59	0.72	0.60
Non-PRR	0.96	0.46	0.63	0.84	0.62	0.71	0.96	0.81	0.88
PRR	0.12	0.80	0.20	0.64	0.85	0.73	0.23	0.62	0.33
*Di-Gr-LL [av]*	0.54	0.63	0.48	0.76	0.76	0.76	0.61	0.77	0.62
Non-PRR	0.95	0.62	0.75	0.81	0.75	0.78	0.97	0.78	0.87
PRR	0.13	0.64	0.22	0.72	0.78	0.75	0.24	0.75	0.36
*SML [PA] [av]*	0.74	0.66	0.69	0.82	0.64	0.6	0.59	0.66	0.61
Non-PRR	0.94	0.97	0.96	0.63	1.0	0.77	0.94	0.88	0.91
PRR	0.53	0.34	0.42	1.0	0.28	0.43	0.24	0.44	0.31
*SML [BNB] [av]*	0.75	0.64	0.68	0.75	0.61	0.56	0.65	0.58	0.60
Non-PRR	0.94	0.98	0.96	0.61	0.98	0.75	0.93	0.97	0.95
PRR	0.56	0.31	0.4	0.89	0.24	0.38	0.38	0.19	0.25
*SML [MNB] [av]*	0.79	0.61	0.65	0.76	0.56	0.48	0.63	0.58	0.59
Non-PRR	0.94	0.99	0.96	0.59	0.99	0.74	0.93	0.97	0.95
PRR	0.64	0.23	0.34	0.94	0.13	0.22	0.33	0.19	0.24
*SML [LR] [av]*	0.67	0.71	0.69	0.83	0.76	0.76	0.64	0.82	0.66
Non-PRR	0.95	0.93	0.94	0.73	0.96	0.83	0.98	0.82	0.90
PRR	0.38	0.49	0.43	0.93	0.56	0.7	0.30	0.81	0.43
*SML [SGD] [av]*	0.66	0.66	0.66	0.81	0.7	0.7	0.66	0.68	0.67
Non-PRR	0.94	0.95	0.94	0.68	0.98	0.8	0.95	0.93	0.94
PRR	0.37	0.37	0.37	0.94	0.43	0.59	0.37	0.44	0.40
*DL [CNN] [av]*	0.78	0.63	0.67	0.77	0.59	0.54	0.58	0.59	0.59
Non-PRR	0.94	0.99	0.96	0.61	0.99	0.75	0.93	0.93	0.93
PRR	0.62	0.27	0.37	0.93	0.20	0.33	0.24	0.25	0.24
*DL [LSTM] [av]*	0.79	0.65	0.69	0.80	0.62	0.58	0.62	0.72	0.65
Non-PRR	0.94	0.98	0.96	0.62	0.99	0.77	0.96	0.88	0.91
PRR	0.63	0.31	0.42	0.97	0.25	0.40	0.29	0.56	0.38
*DL [BERT] [av]*	0.66	0.76	0.69	0.77	0.75	0.75	0.77	0.70	0.73
Non-PRR	0.96	0.89	0.93	0.70	0.89	0.78	0.95	0.97	0.96
PRR	0.36	0.63	0.45	0.84	0.61	0.71	0.58	0.44	0.50

### Appendix A7: F1 scores for ensemble PRR content detection models

This appendix provides a detailed overview of the performance metrics for 330 ensemble PRR content detection models for the document-based test dataset. Specifically, it provides information about the model performance for detection of non-PRR and PRR classes together with the average performance values. Due to the space limitations, the only metric which is provided is F1 scores, which are the harmonic means of precision and recall.

The metrics are provided in Figures 7, 8, 9, 10, 11 and 12. Each figure corresponds to one of the six modes of preprocessing (e.g., no preprocessing or stemming only) and contains information about the performance of the ensemble models based on the combinations of individual models listed on the vertical and horizontal axes.

## Footnotes

1 The presence of such information was identified using the Comparative Agenda project-based dictionary for German language; for more information see Appendix A1).

2 The necessity to rely on universal HTML parsers is a major challenge associated with the use of web-tracking data (i.e., our use case), as well as the related problem of cross-platform automated content analysis. The use of universal parsers results in the higher volume of noise associated with parsing errors (e.g., infection of organic text with malparsed HTML tags), but the only alternative is the use of platform-specific parsers which is not feasible when data come from thousands of platforms (see for more information Appendix A2).
